# 3D Printing of Periodic Porous Metamaterials for Tunable Electromagnetic Shielding Across Broad Frequencies

**DOI:** 10.1007/s40820-024-01502-5

**Published:** 2024-09-03

**Authors:** Qinniu Lv, Zilin Peng, Haoran Pei, Xinxing Zhang, Yinghong Chen, Huarong Zhang, Xu Zhu, Shulong Wu

**Affiliations:** 1 State Key Laboratory of Polymer Materials Engineering, Polymer Research Institute of Sichuan University, No. 24 South Section 1, Yihuan Road, Chengdu, 610065 People’s Republic of China; 2 Baosheng Technology Innovation Corporation Limited, No. 1, Suzhong Road, Baoying County, Yangzhou, 225800 People’s Republic of China

**Keywords:** Polymeric component, 3D printing, Tunable electromagnetic shielding, Periodic porous metamaterials, Honeycomb pore structure

## Abstract

The new-generation electronic components require a balance between electromagnetic interference shielding efficiency and open structure factors such as ventilation and heat dissipation. In addition, realizing the tunable shielding of porous shields over a wide range of wavelengths is even more challenging. In this study, the well-prepared thermoplastic polyurethane/carbon nanotubes composites were used to fabricate the novel periodic porous flexible metamaterials using fused deposition modeling 3D printing. Particularly, the investigation focuses on optimization of pore geometry, size, dislocation configuration and material thickness, thus establishing a clear correlation between structural parameters and shielding property. Both experimental and simulation results have validated the superior shielding performance of hexagon derived honeycomb structure over other designs, and proposed the failure shielding size (*D*_*f*_ ≈*λ*/8 − *λ*/5) and critical inclined angle (*θ*_*f*_ ≈43° − 48°), which could be used as new benchmarks for tunable electromagnetic shielding. In addition, the proper regulation of the material thickness could remarkably enhance the maximum shielding capability (85 − 95 dB) and absorption coefficient *A* (over 0.83). The final innovative design of the porous shielding box also exhibits good shielding effectiveness across a broad frequency range (over 2.4 GHz), opening up novel pathways for individualized and diversified shielding solutions.

**Supplementary Information:**

The online version contains supplementary material available at 10.1007/s40820-024-01502-5.

## Highlights

- The stable periodic porous shielding materials were prepared by combining the strategies of 3D printing and metamaterial design.
- The relationship between porous material structure and electromagnetic interference shielding efficiency (EMI SE) effectiveness was deeply explored, revealing the important structural parameters for realizing tunable EMI SE property.
- The optimized design of the periodic porous shielding box achieves effective EMI shielding in a wide wavelength range (over 2.4 GHz).

**Supplementary Information:**

The online version contains supplementary material available at 10.1007/s40820-024-01502-5.

## Graphical abstract

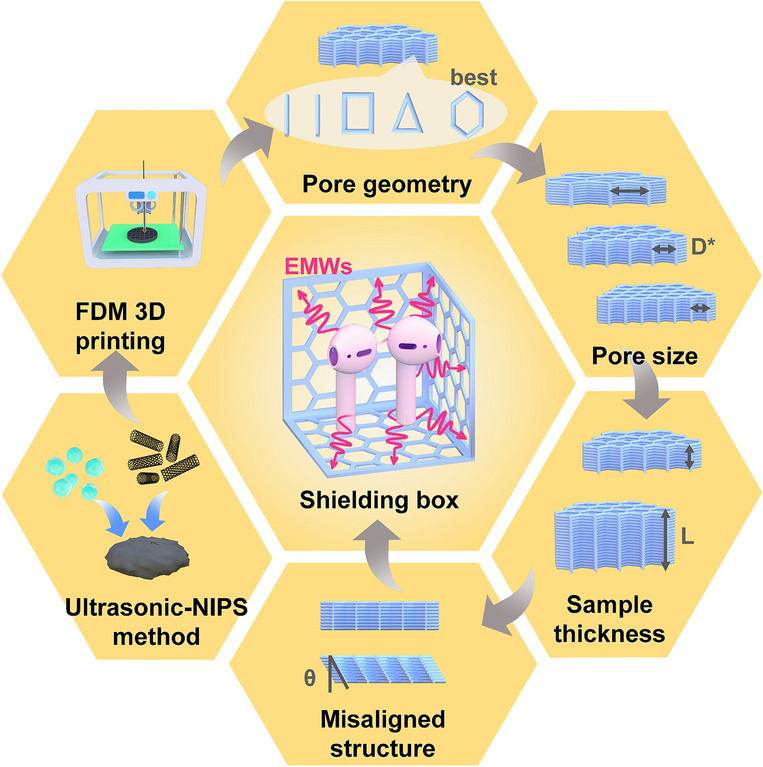

## Introduction

Since the third revolution of science and technology, the rapid evolution of communication and microelectronic equipment based on information technology has significantly enhanced the efficiency of resource optimization, thus catalyzing the transformation and upgrading of the traditional industries [1–5]. Concurrently, the electromagnetic (EM) radiation generated by the working communication and electronic devices exhibit the strong scattering and penetration abilities, thus causing the inevitable damages to human health, the environment, and electronic devices [6–9]. In addressing these challenges, various countries globally have been focusing on developing a series of materials with electromagnetic interference shielding (EMI SE) functions to mitigate the electromagnetic pollution [10–13]. However, the current research tends to concentrate on elevating the values of shielding efficiency, often overlooks the possible implications and challenges when applying the EMI SE materials [14–19].

In the practical service of EMI SE parts, due to the presence of ventilation, heat dissipation, power and various type of signal interfaces upon installation of equipment, instruments and boxes, there are the unavoidable holes and gaps occurring, resulting in electromagnetic leakage that critically impacts the shielding effectiveness of the entire shielding body [20]. Therefore, the effective management and design of more efficient pore structures are very crucial in design of shielding bodies. For sealable seams such as those at instrument and box joints, the conventional treatments include sanding, adding fasteners, and applying electromagnetic sealing gaskets [21, 22]. However, for the permanent gaps like ventilation and heat dissipation holes, it is essential to design and develop some specialized porous shield components with EMI SE properties to overcome the challenges of these specific types of holes and gaps [23].

Thus far, the most commonly used porous shielding parts predominantly consist of metal, such as metal mesh, porous metal plates, and cutoff waveguide vents [22, 24]. Researchers like Alessio Tamburrano have leveraged electromagnetic modeling to assess the shielding efficiency of metal meshes, finding that they are typically more suitable for low-frequency than high-frequency sources [22]. Although the cutoff waveguide vents are suitable for high-frequency field sources, their considerable length-to-diameter ratios require the excessive space. Ener Salinas et. al. simulated and analyzed the electrical contact between seams during the installation of the metal mesh [25]. Their studies showed that even after welding and flange fastening, the metal porous shields need additional sealing for the complete electrical continuity, and such the process may heighten the electromagnetic leakage risks. Consequently, there is a pressing requirement for developing flexible porous shields that can be directly installed in a single step. In addition, with the study being increasingly deepen, the limitations of metal porous shielding materials are further demonstrated [26–28]. These shields, designed basically based on high-pass filter theories for ideal conductors (zero resistance), fall short of adequately elucidating the relationship between electromagnetic waves (EMWs) wavelengths and pore sizes, particularly for non-ideal conductors in the periodic porous shield designs. Consequently, there is an essential need for further refinement of these shielding theories and mechanisms [29, 30]; more specifically, the metal shielding materials have a high specific gravity and are not corrosion-resistant. The shielding mechanism is only based on total reflection [31–34]. In addition, they are usually customized products, which have problems such as single preparation method, long preparation cycle and high cost, and do not align with the current trends toward personalized and diversified product development [35, 36]. Therefore, embracing new structures, technologies and methodologies are crucial for innovating porous EMI SE bodies that cater to personalized and diverse applications [37–39].

3D printing is an additive manufacturing technology based on the principle of dimensional reduction manufacturing [40]. While saving materials, it can realize design and rapid fabrication of various complex structures, including development of porous EMI SE components [41]. Jing et al. prepared LLDPE/GNPs porous EMI SE parts by combining FDM 3D printing with microwave sintering to enhance the part interface. The obtained products showed the excellent mechanical properties and EMI SE performance, achieving the shielding efficiency of up to 32.4 dB in the X-band range [10]. Shi et al. combined FDM 3D printing with the filler local enrichment strategy to prepare the porous components of PLA/GNPs composites based on exploring the printing flow behavior, and the EMI SE efficiency of the printed porous products could reach up to 34.7 dB at different layer thickness [42]. After literature survey, it is easily found that most of the current studies involving porous EMI SE composites mainly focus on improving the shielding efficiency of the composites by considering the content of fillers, the network structure construction, and the morphology control, but seldom carefully explore the relationship between the printing porous structure parameters and the corresponding EMI SE performance.

In order to delve into the influence of holes and seams on EMI SE efficiency and explore effective electromagnetic shielding structures in shielding bodies, in this work we intended to design and fabricate the personalized and multifaceted periodic porous shielding metamaterials through using 3D printing strategy. Firstly, the thermoplastic polyurethane/carbon nanotubes (TPU/CNTs) nanocomposites with uniform dispersion and stable performance were prepared by adopting an innovative method of combining nonsolvent-induced phase separation (NIPS) and ultrasound. Furthermore, fully leveraging the advantages of fused deposition modeling (FDM) 3D printing technology in complicated structure fabrication and personalized manufacturing [43–47], we also innovatively designed and printed the periodic porous flexible shielding metamaterials with multiple shapes, structures and scales. Through simulation and experimental verification, the intrinsic correlations between its structure (pore geometry, pore size, material thickness and pore dislocation configuration) and the overall shielding mechanisms and effectiveness were thoroughly explored and analyzed. More surprisingly, by adjusting structure, the tunable shielding of EMWs at different frequency can be effectively realized. Finally, directed by the above findings, a porous shielding box was accordingly designed and printed, which could effectively realize the wide-frequency range of shielding function. This pioneering investigation could provide novel perspectives and methodologies for designing and developing the porous flexible shielding metamaterials.

## Experimental Section

### Materials

Thermoplastic polyurethane (TPU, Desmopan 365) with a density of 1.230 g cm^−3^, a shore hardness about 65HD (15 s), was provided by Covestro (Germany). Carbon nanotubes (CNTs, NC7000) with a surface area of 250 ~ 300 m^2^ g^−1^ and a volume resistivity of 10^−4^ Ω cm were obtained from Nanocyl S.A., Belgium. N, N-dimethylformamide (DMF), polyvinylpyrrolidone K30 (PVP) and deionized water were purchased from Kelong Chemical Reagent Factory, China.

### Preparation of TPU/CNTs Nanocomposite Filament

The TPU/CNTs nanocomposite filaments were prepared by using ultrasonic dispersion coupled with nonsolvent-induced phase separation (NIPS) method. Firstly, a certain amount of TPU particles were added into 500 mL DMF and stirred at 25 °C for 4 h to dissolve completely. Then, the calculated proportion of CNTs and PVP powders were added to another 500 mL DMF under the effect of combined ultrasound (800 W) and mechanical stirring (500 rpm) at 25 °C for 0.5 h. After the stable CNTs suspension was formed, it was mixed again with the well-dissolved TPU solution in DMF at 25 °C for another 1 h under ultrasound and stirring so as to obtain a stable suspension of TPU/CNTs mixtures dispersed in DMF. The resultant TPU/CNTs mixture suspension was added dropwise to a large amount of deionized water for solution exchange. After multiple washes for removal of PVP-K30 and DMF, the finally obtained mixed suspension was filtered and dried at 80 °C for 48 h to obtain the TPU/CNTs composite blocks. After that, the obtained TPU/CNTs compounds were extruded in a single screw extruder (RM-200C, Harbin HAPRO Electric Technology Co., Ltd. China) to prepare the FDM 3D printing filaments with a diameter of 1.75 ± 0.05 mm. The extrusion temperature and extrusion speed were set at 190–230 °C and 20 rpm, respectively. For convenience, the above prepared TPU/CNTs nanocomposite sample with n wt% CNTs was named L-n. Similarly, the pure TPU filaments were also prepared in the RM-200C extruder at the same extrusion conditions. The formulations for various samples involved in this paper are included in Table S1.

### FDM 3D Printing of TPU/CNTs Nanocomposite

The designed model was digitally sliced using Simplify 3D software, and the resultant G-Code file was imported into the printer (German RepRap X350pro, Feldkirchen, Germany) for printing to produce the corresponding 3D printed TPU/CNTs functional parts. The detailed printing parameters are listed in Table S2.

### Characterization

Ultraviolet–visible near-infrared spectrophotometer (UV–Vis-NIR) spectra were measured by the SHIMADZU UV-3600 (Japan) with a wavelength of 200–1200 nm and a resolution of 0.1 nm. The zeta-potential and nanoparticle size of the suspensions were tested using a nanoparticle size potential analyzer (Zetasier Nano-ZS, Malvern, UK). Scanning electron microscope (SEM) images were observed by FEI INSPECT F (USA) with an accelerated voltage of 5–20 kV. The rheological properties were assessed using a rotational rheometer (AR2000ex, TA Instruments, USA) with a frequency range of 0.01–100 Hz and at a fixed strain of 1%. Mechanical properties were tested using Instron 5576 (INSTRON, USA) on five samples in each group, and results were averaged, where the compression strength test is performed at a rate of 10 mm/min under 70% strain, and the test of compression permanent deformation rate is conducted by being kept at 23 °C under 25% strain for 72 h. Electrical conductivity tests were performed using a four-probe instrument (FT-331, Guangzhou Four-Point Probe Technology Co., Ltd, China). The infrared thermal imaging photographs of the 3D printed samples were captured by a Testo infrared thermal imager (Testo 870–2). Electromagnetic intensity was tested using an electromagnetic field tester (LZT-6200, China). The EMI SE performance (S-parameter, including *S11*, *S21*, *S12* and *S22*) were characterized by a vector network analyzer (VNA, Agilent N5230, USA) with length × width dimension of 22.9 × 10.2 mm^2^ (8.2–12.4 GHz) and 15.8 × 7.9 mm^2^ (11.9–18.0 GHz), and 0.0 ~ 10.0 mm thickness. The electromagnetic parameters (including T*T*, *R*, *A*, SE_T_, SE_R_, SE_A_ and SE_m_) were calculated using the following equations [48–51]:

1 $$R = \left| {S_{11} } \right|^{2} ,\,T = \left| {S_{21} } \right|^{2}$$

2 $$A = 1 - R - T$$

3 $${\text{SE}}_{{\text{R}}} = - 10\log \left( {1 - R} \right)$$

4 $${\text{SE}}_{{\text{A}}} = - 10\log \left( {T/\left( {1 - R} \right)} \right)$$

5 $${\text{SE}}_{{\text{T}}} = {\text{SE}}_{{\text{A}}} + {\text{SE}}_{{\text{R}}} + {\text{SE}}_{{\text{m}}}$$

where *T* is the transmission coefficient, *R* is the reflection coefficient, *A* is the absorption coefficient, SE_T_ is the total EMI SE, SE_R_ is the reflection of EMWs, SE_A_ is the absorption of EMWs, and SE_m_ is the multiple internal reflection of EMWs. Generally, SE_m_ can be ignored if SE_T_ ≥ 10 dB.

## Results and Discussion

### Preparation and Characterization of TPU/CNTs (L-n) Nanocomposites and Filaments

**Fig. 1:**
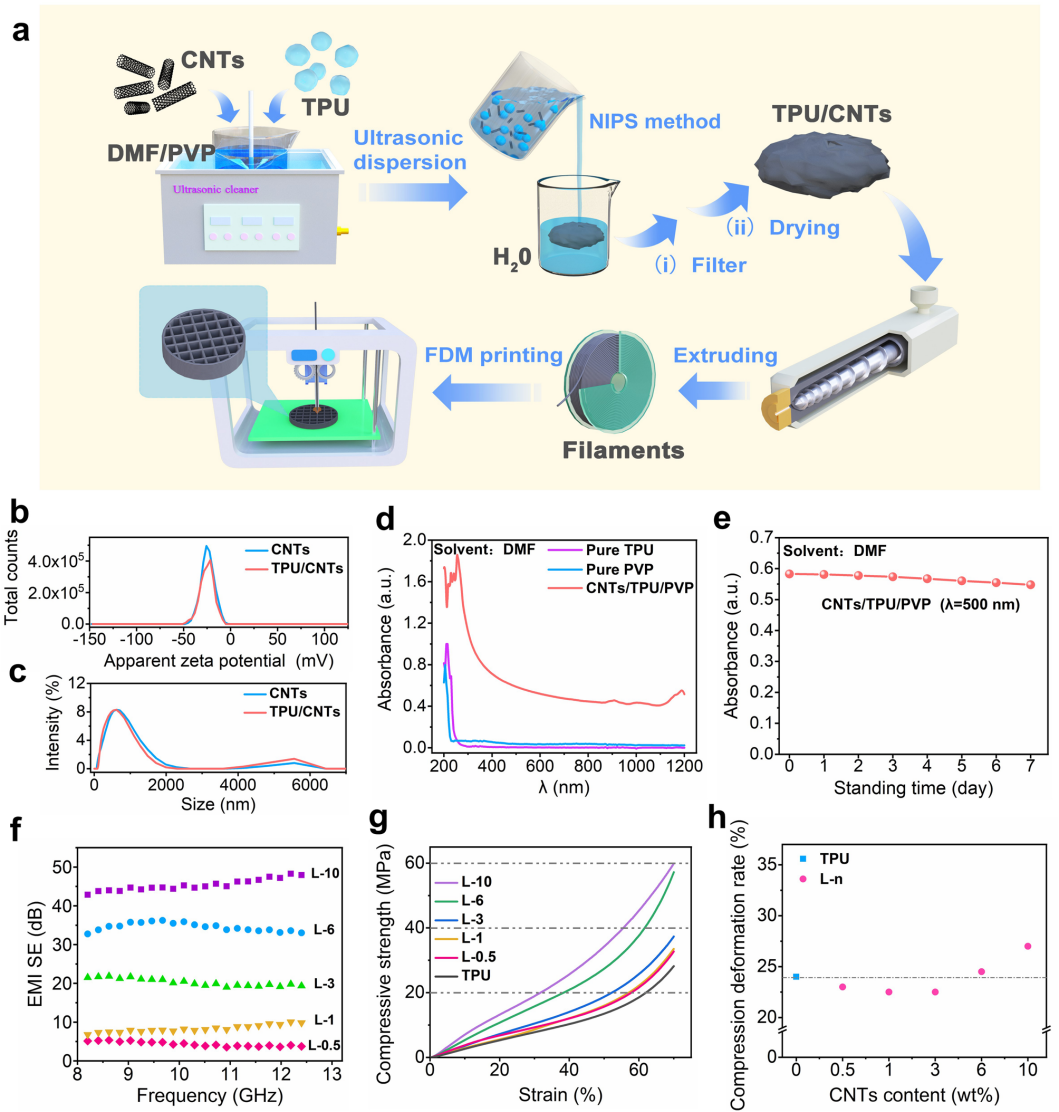
**a** Schematic diagram for preparation of TPU/CNTs printing filaments and the 3D printing process. The characterization of CNTs and TPU/CNTs suspension, including **b** Zeta potential, **c** particle size distribution and **d** UV–Vis-NIR spectrum; **e** CNTs stability characterization by using UV–Vis-NIR spectrum at 500 nm wavelength; **f** EMI SE of TPU/CNTs composite filament, **g** compressive strength versus strain curves and **h** the compression deformation rate of L-n composites with different CNTs content

As shown in Fig. 1a, the TPU/CNTs nanocomposite filaments were prepared by using ultrasonic dispersion coupled with nonsolvent-induced phase separation (NIPS) method. The good dispersion of the filler can bring more uniform and stable performance for the composite material, which can provide accuracy and reproducibility for revealing the relationship between pore structure and EMWs [42, 52]. Therefore, ensuring effective dispersion of CNTs is critical throughout the all phases of fabricating TPU/CNTs nanocomposite materials (including ultrasonic dispersion stage, solvent exchange stage, and composite material forming stage). As shown in Fig. 1b, c, the Zeta potential and particle size analyses indicate that the Zeta potentials of CNTs and TPU/CNTs suspensions were impressively high, which are at 25.7 and 21.6 mV, respectively, and they both possess the particle size below 1200 nm. This confirms the absence of large particles or agglomerates, signifying an efficient dispersion of CNTs. Additionally, the UV–visible near-infrared spectroscopy used to assess the stability of CNTs suspension demonstrates that the absorbance of TPU and PVP in the range of 200–1200 nm wavelength does not interfere with that of CNTs (Fig. 1d). Therefore, the absorbance at 500 nm was selected for the stability evaluation (Fig. 1e). The results revealed that the absorbance of CNTs decreases marginally (only by 5%) after seven days standing, showing the remarkable stability of the CNTs suspension. In addition, the excellent dispersion and stability of CNTs in the TPU matrix were also demonstrated by various tests such as filler settlement experiment, and fractured cross-section morphology observation (Figs. S1–S3). Obviously, the approach by combining the ultrasonic dispersion with the NIPS strategies could effectively overcome the challenges of filler agglomeration and sedimentation, which is often encountered in the directly melt compounding and conventional solution evaporation methods.

The TPU/CNTs compounds were extruded in a single screw extruder to prepare TPU/CNTs composite filaments. The SEM images of the appearance and fracture cross section of the TPU/CNTs filament (Figs. S4 and S5) indicate that the prepared filaments are smooth and have uniform diameter (1.75 mm), which is crucial for ensuring stable extrusion of the filament during the printing process. In addition, the rheological and electrical conductivity data show a uniform distribution of fillers within the composite and also a continuous improvement in the conductive network formation (Figs. S6 and S7). The excellent conductivity would generally result in excellent EMI SE properties for materials [29, 53, 54], and therefore the shielding efficiency of the L-n samples (thickness of 2 mm) was determined. The results are shown in Fig. 1f. As can be seen, an increase in CNT content also leads to a corresponding increase in shielding effectiveness. In particular, L-3 sample achieves a shielding effectiveness of 20 dB, thus meeting the commercial EMI SE standard. Furthermore, the shielding effectiveness of the L-6 and L-10 sample impressively reached 35 and 46 dB, respectively, which can effectively block more than 99.9% of EMWs.

As shown in Fig. 1g, h, the compressive strength and compressive deformation of the L-n material and pure TPU were further measured. The compressive modulus was found to increase with rise in the content of CNTs. At a 70% strain, the compressive stress for L-6 and L-10 sample reaches 57.5 and 59.9 MPa, which represents a remarkable enhancement of 105% and 114%, respectively, compared to 28 MPa compressive stress of pure TPU (Fig. 1g). The superior compressive strength is attributed to the rigidity imparted by the CNTs, which could restrict the molecular chain movements to a certain degree and hence improves the mechanical strength of materials. Additionally, the compressive deformation rate *C* can be calculated using the following formula [55]:

6 $$\begin{array}{*{20}c} {C = \frac{{t_{0} - t_{1} }}{{t_{0} - t_{2} }}} \\ \end{array}$$

where *t*_*0*_ is the original height, *t*_*1*_ is the height after recovery, and *t*_*2*_ is the height after compression.

After calculation, the permanent compression deformation rate of pure TPU is found to be 24%. In the case of the L-n samples, this deformation rate demonstrates a firstly slight decrease and then a small increase tendency with increase in CNT content, culminating in L-10 sample with the maximum deformation rate of 27%. The above results show that L-10 and pure TPU have a very close deformation rate (only 3% difference), indicating that the deformation rate of the prepared TPU material is relatively low. Obviously, the prepared TPU/CNTs L-10 filaments have an outstanding comprehensive performance, including excellent EMI SE, good mechanical property and relatively low deformation rate, and thus lay a good basis for preparation of the high-performance electromagnetic shielding parts with complicated shapes and structures by using FDM 3D printing technology.

### 3D Printing Periodic Porous Structure to Tailor EMI SE Efficiency

#### Effect of Pore Shape on EMI SE Efficiency

**Fig. 2:**
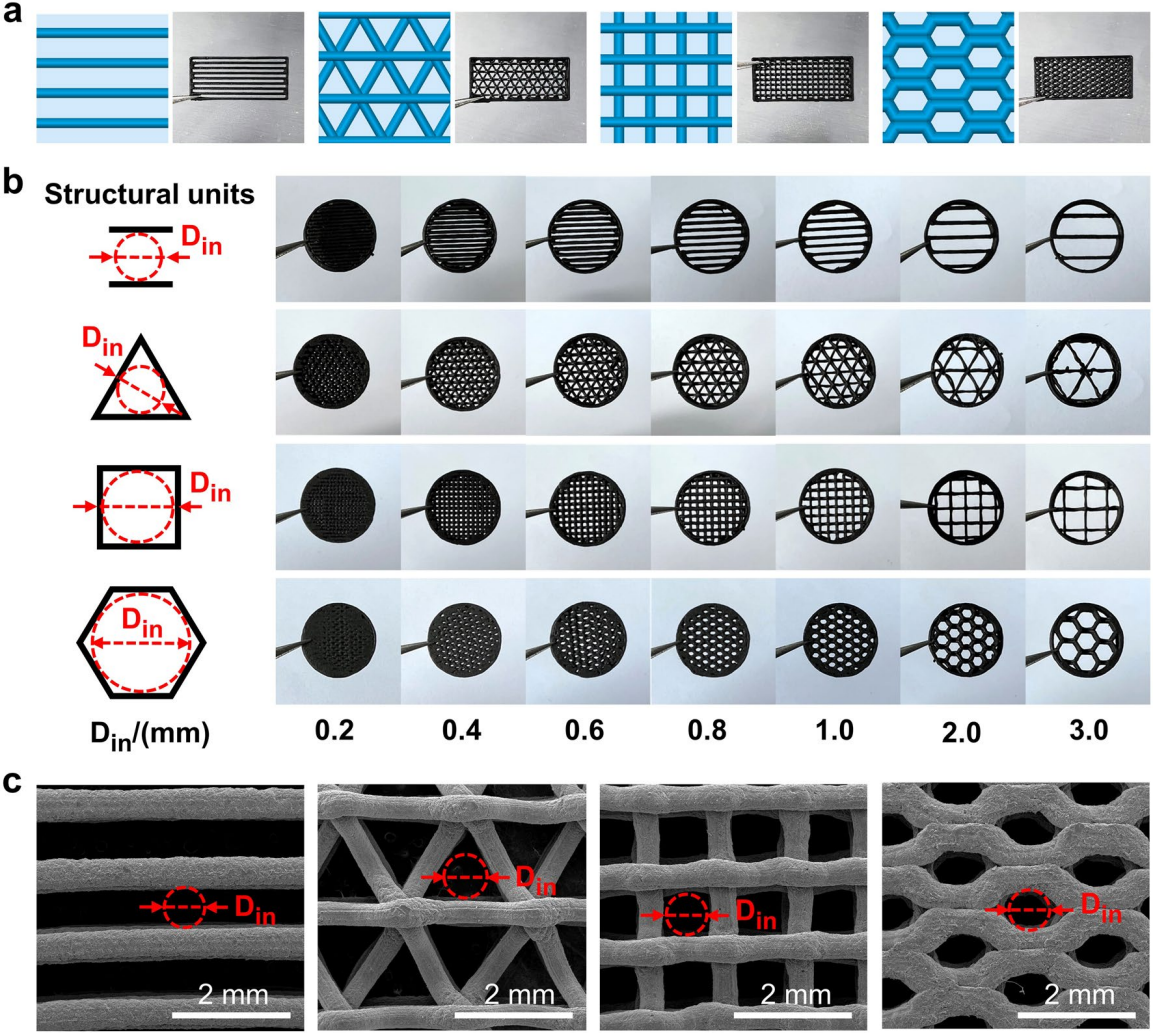
**a** FDM printing models and the optical images of printed parts (dimensions of 22.9 × 10.2 × 2.0 mm^3^) with different periodic pore units; **b** Optimal images of the printed different periodic pore structure units (assembled in circular porous part with diameter 12 mm and thickness 2 mm) with different inscribed circle diameter (*D*_*in*_); **c** SEM images of 0.8 mm *D*_*in*_ size samples with different pore units

EMWs leakage is influenced by a spectrum of factors including the attributes of the electromagnetic irradiation source, the frequency of the electromagnetic field, and the spatial relationship and dimensional characteristics of apertures or gaps relative to the shielding body and the source [22, 56]. Considering the consistent nature of EMWs sources in specific devices and their established distances from these sources, the strategies for mitigating EMWs leakage was predominantly performed to modify the geometry and dimension of the aperture or gap. Within the realm of two-dimensional geometry, generally the triangular, square and hexagonal unit cell can easily constitute a plane using a simple repeating method. In light of the above geometric principle, in this study, we integrated different periodic porous structure unit (including linear (seam), triangular, square and honeycomb (hexagon) configuration), and further employed the FDM 3D printing strategy to realize preparation of porous EMI SE metamaterials with various array patterns, as shown in Fig. 2a–c. These periodic porous structures can easily and naturally partition a two-dimensional plane as required, and the printed periodic porous structures also exhibit high accuracy and smoothness. As indicated in Fig. 2, the *D*_*in*_ used here represents the inscribed circle diameter of different unit pore structure printed (the diameter of the inner circle). For the linear unit pore structure, the *D*_*in*_ is the distance between the two neighboring parallel lines. The determination of *D*_*in*_ size of the designed different unit pore structure is schematically illustrated in Fig. 2b. In order to scientifically characterize the dimension of the different unit pore structure, the equivalent diameter (D^*^), as well as the circumscribed circle diameter (*D*_*out*_) (the diameter of the outer circle), is used. The determination of D^*^ and *D*_*out*_ is explained in Sect. 3.2.2 in this paper.

**Fig. 3:**
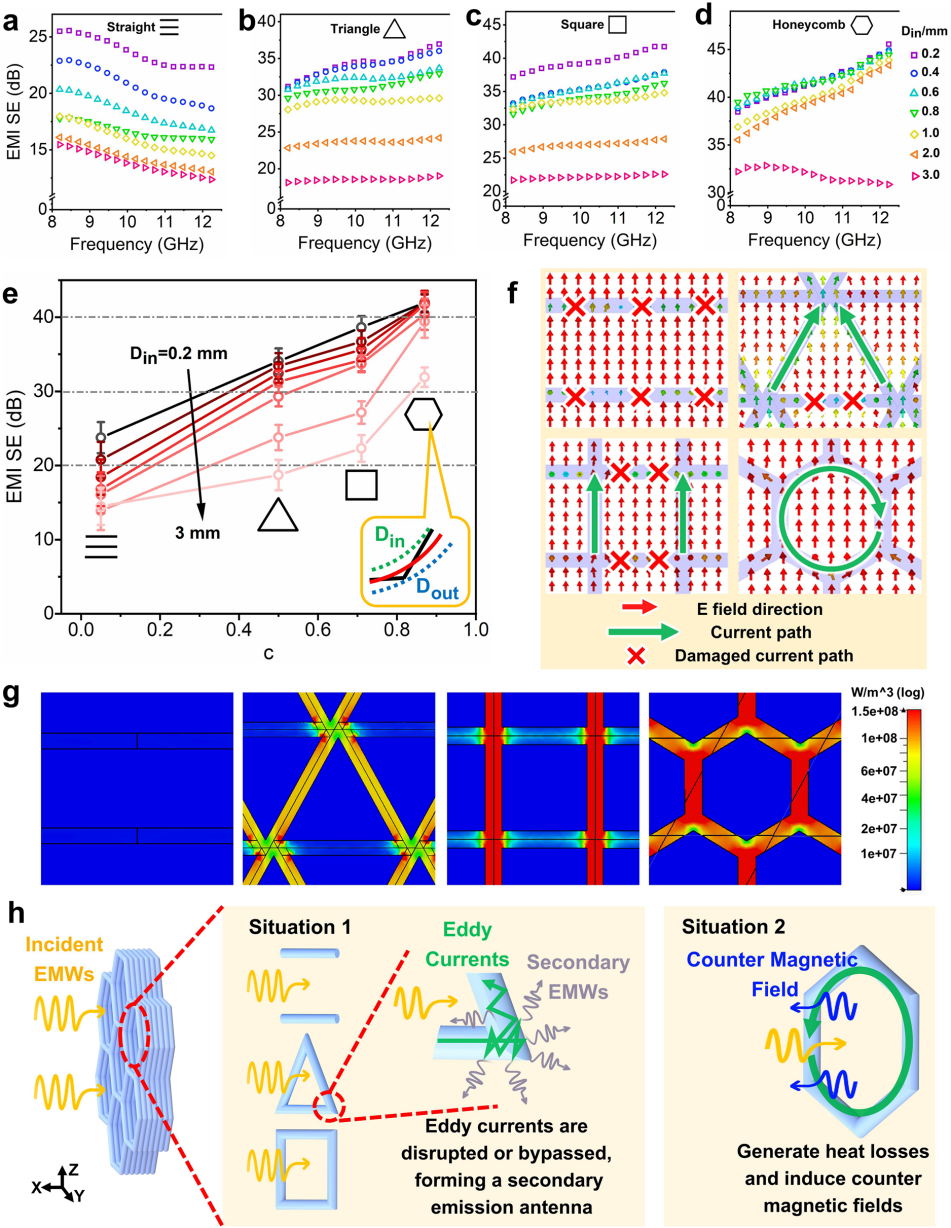
EMI SE of printed samples with various pore structure unit at different *D*_*in*_ including **a** straight, **b** triangle, **c** square and **d** honeycomb; **e** EMI SE versus pore factor *c* of corresponding printed samples; the simulation results of **f** electric-field distribution and **g** power loss density of 2 mm *D*_*in*_ sample with different pore structure unit; **h** schematic representation of the corresponding shielding mechanism

Figure 3a-d presents a comparative study of the EMI SE efficiency across four different periodic structure unit cells in the range of size *D*_*in*_ = 0.2–3 mm within the X-band frequency range. It becomes apparent that the increase in the pore dimension (*D*_*in*_) would proportionally increase the EMWs leakage, thus negatively affecting the EMI SE efficiency of the corresponding structure. Here, it should be noted that in Fig. 3d, the shielding effectiveness of honeycomb pore structure becomes very close when the *D*_*in*_ size is in the range of 0.2–0.6 mm. The reason for this could be possibly explained by the used honeycomb pore structure itself, which could much more effectively enhance the EMI SE performance of the printed part than the involved other pore structure, particularly in the large* D*_*in*_ size range. However, for the small pore size, the increasing degree of EMI SE efficiency would be very limited. The involving essential reason requires the further investigation later. In order to facilitate a more detailed comparison of shielding efficiency among these structures, the porosity factor *c* = *D*_*in*_/*D*_*out*_(0 ≤ *c* ≤ 1) is introduced, denoting the ratio between the diameters of the inscribed (*D*_*in*_) and circumscribed (*D*_*out*_) circles within the structural unit. The evolution of *c* value with the different unit pore structure is shown in Fig. S8 and also the detailed illustrations are also included in Supplementary Material. Obviously, the shape of the pore unit is closer to a circular one, and the value of *c* more approaches 1. Illustrated from Fig. 3e, there is a discernible correlation between the porosity factor *c* and the shielding performance, i.e., the higher the *c* value, the higher the EMI SE efficiency. Consequently, the honeycomb structure achieves the highest EMI SE efficiency (*c* = 0.87, 33–43 dB), outperforming the square (*c* = 0.71, 22–38 dB) and triangular (*c* = 0.5, 18–33 dB) unit, while the linear structure shows the lowest one, offering the least shielding capability (c ≈ 0, 13–23 dB).

The electromagnetic simulations of four different structures with identical size were conducted, and the near-field electrical field distributions are shown in Fig. 3f, where the detailed parameters and the related simulation processes are included in the Supplementary Material with Table S3. There are different changes occurring in the electrical fields within the XY plane under the shielding effect. This phenomenon arises from the resistance loss mechanism inherent being in EMI SE: when EMWs interact with conductive materials, they would induce changes in carrier currents and eddy currents, which in turn change the induced electric fields, thus converting electromagnetic energy into internal energy. The linear, triangular, and square structures would readily intersect paths of eddy currents due to their smaller porosity factor *c* and pronounced angular edges, profoundly disrupting the distribution of induced electric fields within the shield. According to electromagnetic field theory [21, 23], if the integrity of the induced eddy current is compromised, the damaged region would act as a secondary emitting antenna. This results in energy being randomly emitted into the shielding body, thus significantly impairing its shielding effectiveness. Conversely, the honeycomb structure with a similarly circular shape allows for a more natural alignment of electrical fields along its filament framework, thus preserving the stability of induced eddy currents. Furthermore, the parameter of Power loss density in the simulations serves to quantify the electromagnetic power dissipated within lossy materials (illustrated in Fig. 3g). Under a uniform electromagnetic power distribution, the linear structure, due to its discontinuous conductive nature, generally struggles to form the effective induced current pathways under a condition of electromagnetic influence, resulting in the least electromagnetic loss. The sharp areas of triangular and square structures would compromise the completeness of current pathways, leading to rise of electromagnetic losses in these angular zones, and diminished losses in other smoothing areas. Obviously, the integrated and smooth closed-loop design of the honeycomb structures helps maximize the lossy EMWs intensity. These shielding mechanisms are schematically illustrated in Fig. 3h. In order to better understand the influence of different pore structure (linear, triangular, square and honeycomb structure) on the induced eddy currents and electrical fields, and the related mechanism, the detailed explanations and illustrations are also provided in Supplementary Material (including Fig. S9).

#### Effect of Pore Size on EMI SE Efficiency

**Fig. 4:**
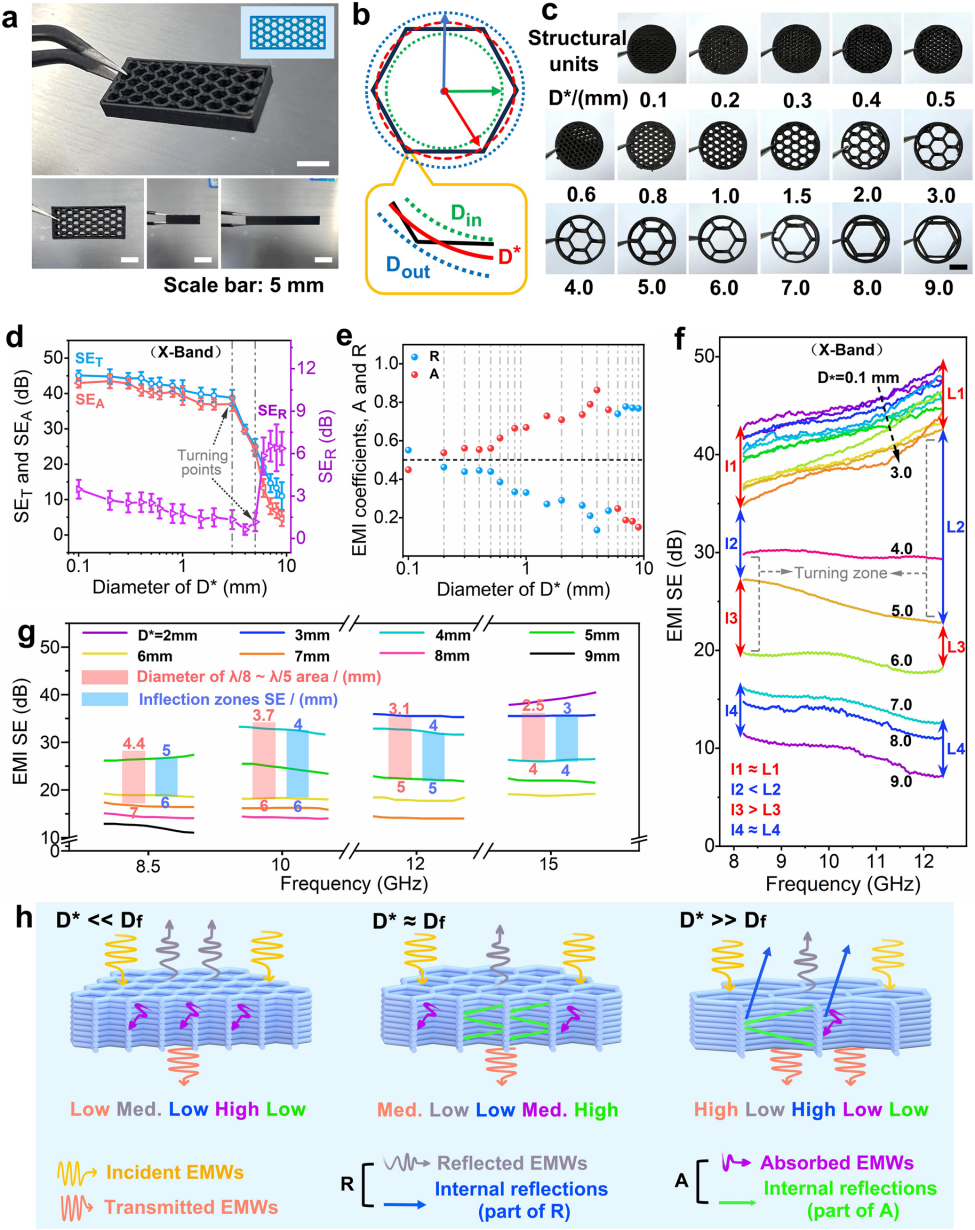
**a** Optical images of honeycomb sample, **b** schematic comparison of parameters *D*_*in*_, *D*_*out*_ and D^*^. **c** Optical images of honeycomb structural units with different D^*^ dimensions. **d** Average electromagnetic parameters (SE_T_, SE_A_, and SE_R_), **e** absorption coefficient *A*, reflection coefficient *R* (at *L* = 2 mm thickness) and **f** overall EMI SE effectiveness in the X-band for printed samples of different D^*^ sizes. **g** EMI SE at 8.5, 10, 12, and 15 GHz for printed honeycomb samples with different size of D^*^ and **h** schematic representation of the corresponding shielding mechanism

In order to further evaluate the shielding mechanisms at various pore dimension, the honeycomb structure was strategically selected as the fundamental pore structure for fabrication of porous EMI SE components with an equivalent diameter D^*^ in the range of 0.1 ~ 9 mm (illustrated in Figs. 4a-c and S10), where D^*^ is defined as:

7 $$\begin{array}{*{20}c} {D^{*} = \sqrt {\frac{{D_{{{\text{in}}}}^{2} + D_{{{\text{out}}}}^{2} }}{2}} } \\ \end{array}$$

where *D*_*in*_ and *D*_*out*_ are the inscribed circle diameter and circumscribed circle diameter, respectively. The Supplementary Material, Table S4, expounds the relationship between D^*^ and the respective *D*_*in*_ and *D*_*out*_ for the four distinct structures. Notably, D^*^ emerges as a more accurate indicator of the true dimensions of an individual honeycomb structure in comparison with *D*_*in*_. Figure 4d-e presents the shielding efficiency parameters (SE_T_, SE_A_ and SE_R_) and EMI coefficients (*A* and *R*) in the X-band. The trends in these parameters basically diverge into two distinct phases: at D^*^ < 3–5 mm, SE_T_ slowly diminishes with increasing D^*^, SE_R_ is less than 3 dB, and the maximum absorption coefficient *A* exceeding 0.85, indicating that the shielding effectiveness is dominated by absorption loss. In contrast, for D^*^ > 3–5 mm, SE_T_ rapidly decreases with increasing D^*^, the reflection coefficient *R* reaches its minimum near D^*^≈5 mm before experiencing a significant enhancement. After that (D^*^≈5 mm), the shielding mechanism is mainly controlled by the reflection loss, and the absorption coefficient *A* decreases significantly. This variation could be attributed to the possible interaction between the D^*^ size at the critical point of shielding effectiveness (3–5 mm) and the wavelength *λ* of the EMWs, which imparts the EMWs different selectivity when passing through different sized pores.

In order to verify this supposition, the shielding performance was further dissected into eight specific regions, as depicted in Fig. 4f. These regions are segmented into four sections including *l1*, *l2*, *l3*, and *l4* at 8.2 GHz, along with another four sections including *L1*, *L2*, *L3*, and *L4* at 12.4 GHz. The analysis reveals four distinct interrelations: (1) When D^*^ is less than 3 mm, *l1* and *L1* are almost identical, reflecting a consistent and gradual decline in EMI SE effectiveness throughout the X-band; (2) At D^*^ of approximately 3–5 mm, *l2* is obviously lower than *L2*, being featured with a steep decrease in EMI SE effectiveness, especially pronounced in the higher frequency range (12.4 GHz); (3) At D^*^ of approximately 5–6 mm, *l3* surpasses *L3*, indicating a continuous sharp decline in EMI SE effectiveness with rise of D^*^, particularly in the lower frequency range (8.2 GHz); (4) At D^*^ of beyond 6 mm, *l4* is equivalent to *L4*, the EMI SE effectiveness shows the obvious decrease tendency across the X-band. This indicates that the pore sizes at the turning points of EMI SE effectiveness are distinct for different frequency range, with the D^*^ size at the turning point in the low-frequency domain (5–6 mm) being considerably larger than in the high-frequency spectrum (3–5 mm). Correlating the D^*^ size at these inflection points with the corresponding EMWs wavelengths in the range of 8.2 ~ 12.4 GHz (i.e., in the range of approximately 36 ~ 24 mm), it becomes apparent that the pore sizes at these points comply with such a relationship of D^*^≈*λ*/8 − *λ*/5. Hereby, the investigation was further expanded to several other frequency bands (8.5, 10.0, 12.0, and 15.0 GHz) to substantiate the above finding, as depicted in Fig. 4g. As can be seen, the inflection zones of EMI SE effectiveness for these bands (marked in blue) are consistently located within the *λ*/8 − *λ*/5 region (marked in red). Consequently, the equivalent size D^*^≈*λ*/8 − *λ*/5 in multi-porous shields is defined as the critical failure shielding size *D*_*f*_. Below this threshold *D*_*f*_, the EMWs struggle to directly penetrate the pores, and would undergo the multiple reflections and absorptions within the pores. Also, the shielding effectiveness gradually decreases with increase in pore size, primarily being dominated by absorption loss. In contrast, at or above *D*_*f*_, the EMWs can directly pass through the pores, and only a minimal portion of EMWs is attenuated by the outer surface of material. The proportion of EMWs loss within the pores continuously decreases. As such, the EMI SE value sharply decreases with increasing pore size, and the shielding mechanism is dominated by reflection loss. The related shielding mechanisms are well schematically illustrated in Fig. 4h. In addition, Table S5 presents a comparison of EMI SE between this work and the other studies involving carbon-based functional materials, and exhibiting the advantage of our work.

#### Effect of Porous Part Thickness on EMI SE Efficiency

**Fig. 5:**
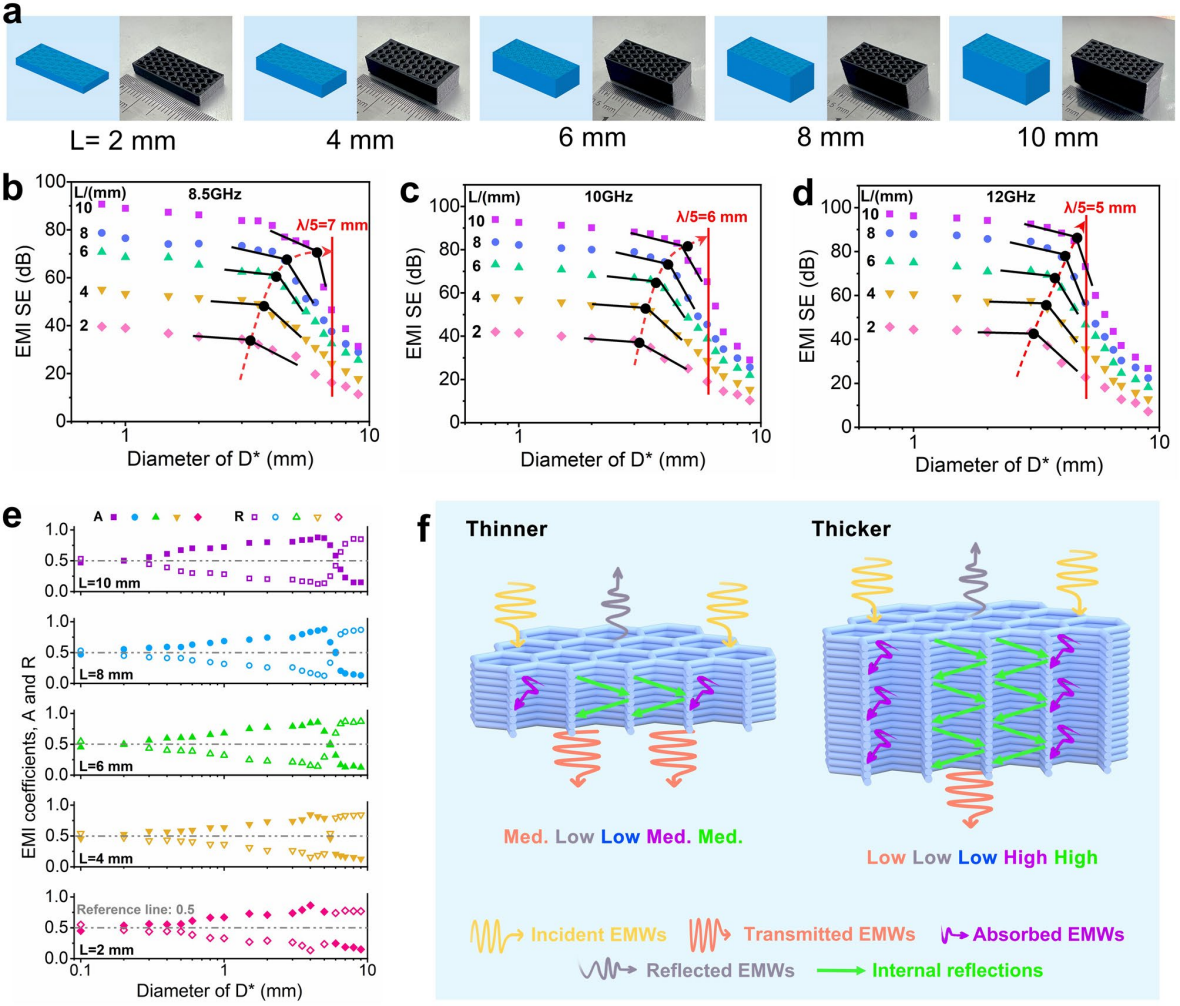
**a** FDM printing models, optical images of printed samples with different thickness. The EMI SE at **b** 8.5, **c** 10, **d** 12 GHz and the **e** absorption coefficient *A* and reflection coefficient *R* of printed samples with different thickness. **f** Schematic representation of the corresponding shielding mechanism

As depicted in Fig. 5a, the fabrication of multi-porous honeycomb EMI SE components with different thicknesses *L* (2, 4, 6, 8, and 10 mm) was further performed, followed by an evaluation of their EMI SE efficiency within the X-band range, as demonstrated in Fig. 5b–d. It was observed that the shielding efficiency in each frequency band progressively augments with increasing thickness. Specifically, at D^*^ = 0.8 mm, the mean shielding efficiency at three frequencies (8.5, 10, and 12 GHz) is advanced from 39.6, 45.5, and 49.3 dB at a thickness of *L* = 2 mm, to an impressive 90.7, 93.9, and 97.1 dB at *L* = 10 mm, respectively. Generally, the shielding effectiveness of as high as 90 dB, which would effectively block 99.9999999% of EMWs, significantly surpasses the stringent standards required for military applications (60 dB) and TEMPEST shielding devices (80 dB), thus rendering the 3D printed composite suitable for use in shielding chambers or enclosures. The reason for the above change is that, on one hand, the increase in the thickness of the printed component would lead to the increase in the number of the total effective shielding pores (also the total pore size), thus resulting in the enhancement of the EMI SE performance; on the other hand, the variation of shielding efficiency with thickness is also correlated to the involved critical failure size *D*_*f*_ of shielding. With the continuous increase in thickness, the critical failure size *D*_*f*_ of the shielding effectiveness also gradually increases (shifting toward *D*_*f*_ ≈ *λ*/5 from its initial range of *D*_*f*_ ≈ *λ*/8 − *λ*/5, Fig. 5b–d), thus expanding the effective shielding pore size of the shielding component (the reason for such the change is explained in Supplementary Material by combining with Fig. 4h). Furthermore, as shown in Fig. 5e, for products with pore size marginally less than *D*_*f*_, the absorption coefficient *A* across different thickness is strikingly high, averaging at above 0.83, e.g., at a thickness of 10 mm and near the pore size D^*^ = 4 mm, the absorption coefficient *A* could reach an impressive 0.87 (the *A* value is greater than 0.5 at pore size D^*^ < 6 mm). The shielding mechanism is dominated by the absorption loss. This is because when the pores are slightly smaller than the *D*_*f*_ size, apart from a portion of EMWs being directly lost at the material surface, a significant number of EMWs would enter the interior of these pores. This would lead to further loss of EMWs within the pores, thus increasing the overall absorption loss of shielding component. Also, in the range of less than *D*_*f*_ size, the increase in pore size would result in a diminished surface area of the components, subsequently reducing the reflection loss at the composite surface. As a result, the absorption coefficient *A* of the shielding component is effectively maximized by considering the above factors. Obviously, the combination Fig. 5f with Fig. 4h could provide a good explanation for the above shielding mechanism.

#### Effect of Pore Dislocation Configuration on EMI SE Efficiency

**Fig. 6:**
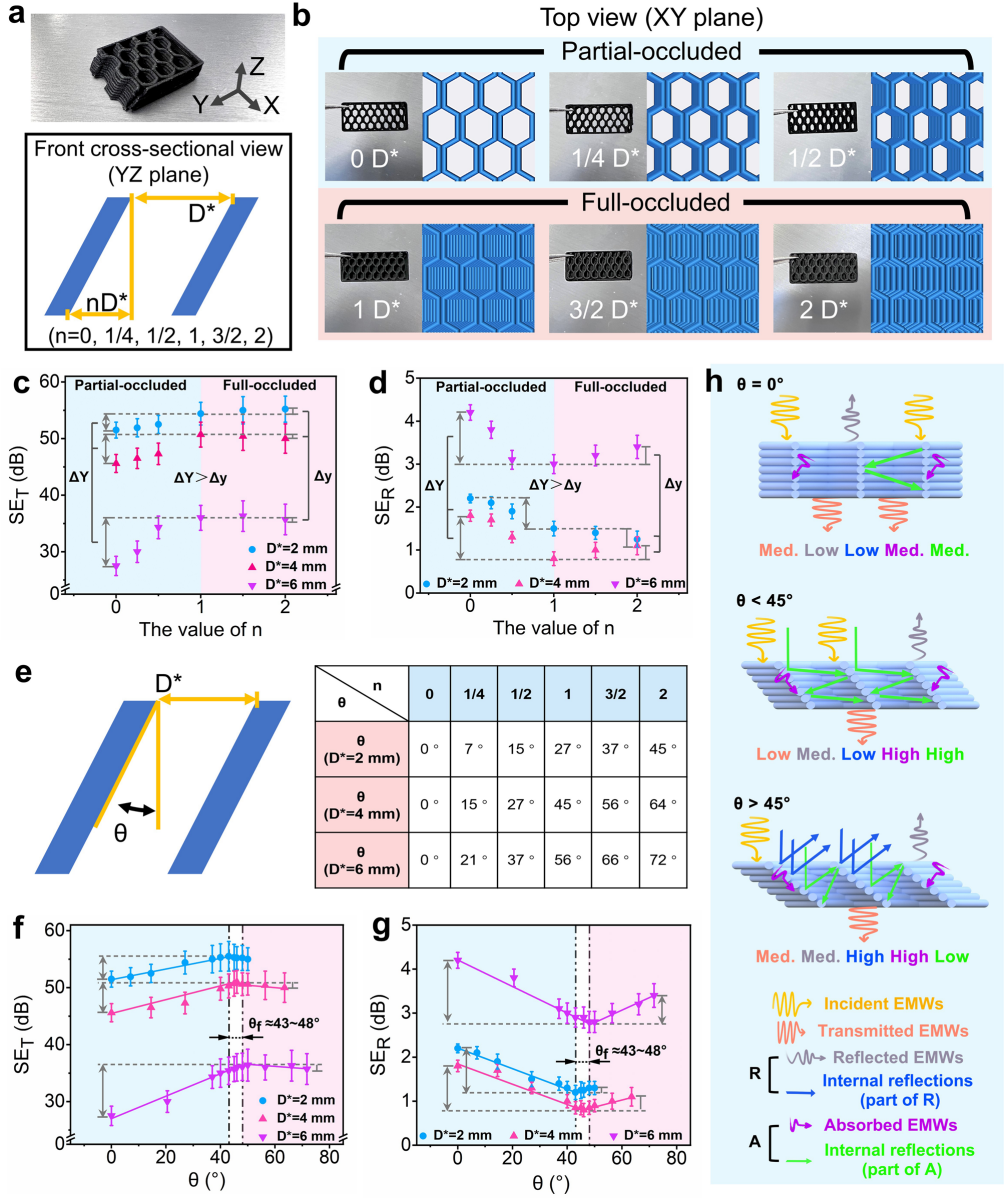
**a** Optical image of the misaligned structure and a schematic diagram of its front view cross section (YZ plane); **b** top view (XY plane) of the FDM printing models and optical images; **c** SE_T_ and **d** SE_R_ of printed samples with different misaligned structure; **e** schematic diagrams and values of different inclined angles *θ*; **f** SE_T_ and **g** SE_R_ of printed samples with different inclined angles *θ*; **h** schematic representation of the corresponding shielding mechanism

Finally, the related shielding mechanisms of multi-porous components with dislocation configuration were carefully investigated and discussed. Figure 6a, b depicts these components with different degrees of dislocation, showing the structure of different samples with partial (0 D^*^, 1/4 D^*^, and 1/2 D^*^) and full occlusion (1 D^*^, 3/2 D^*^, and 2 D^*^). Figure 6c shows the shielding effectiveness results of these dislocated multi-porous components across different D^*^ dimension (2, 4, and 6 mm). The findings reveal that changes in the occlusion ratios of the unit pores would also lead to the significant variations in SE_T_ values of the corresponding samples. Under the partial-occluded conditions (*n* is in the range of 0–1), there is an increase trend in SE_T_ value with increasing occlusion ratio. Conversely, in the full-occluded state (*n* is in the range of 1–2), although the SE_T_ values vary with different sample of different D^*^ size, the overall variation in SE_T_ remains relatively small. The corresponding SE_R_ result spectra, as shown in Fig. 6d, exhibit a similar behavior. Under the partial-occluded conditions, the SE_R_ values show the significant changes, decreasing with increasing occlusion ratio, while under full-occluded conditions, the SE_R_ changes are relatively smaller. These variations in SE_T_ and SE_R_ suggest the feasibility of tuning EMI SE parameters through strategic regulation of the occlusion ratio in scenarios involving partial-occluded pores. The further investigation indicates that although there are varying trends in SE_T_ and SE_R_, the different dislocation structures can be more clearly and conveniently represented and analyzed by the pore inclined angle (Fig. 6e). Accordingly, the inclined angle *θ* was introduced, and the electromagnetic parameters (SE_T_ and SE_R_) of different inclined angle *θ* (including 40°, 43°, 46°, 48°, and 50°) was further supplemented, which were used to investigate the influence of inclined angle *θ* (dislocation structure) on the electromagnetic parameters, as shown in Fig. 6f, g. As can be seen, at this condition, the electromagnetic parameters across different D^*^ dimension demonstrated a consistent change trend. In terms of SE_T_ values, for the printed part with different D^*^ size, an increase tendency was observed with increasing *θ* in the range of *θ* < 43° ~ 48°. The effect of *θ* on SE_T_ is more pronounced for the larger D^*^ size, e.g., the component of D^*^ = 6 mm shows the greatest influence, displaying a fluctuation as high as 10 dB, and however, the component of D^*^ = 4 and 2 mm exhibits a variation of around 6 and 4 dB, respectively. When *θ* > 43° ~ 48°, all components exhibit an inflection point in SE_T_, and an initial decrease tendency is revealed. Similarly, SE_R_ also shows a inflection point around *θ* ≈ 43° ~ 48°, which decreases with increase in *θ* up to 43° ~ 48°, and then gradually increases beyond *θ* of 43° ~ 48°. Obviously, the change tendencies of SE_T_ and SE_R_ imply that there is a critical inclined angle *θ*_*f*_ ≈43° ~ 48° existing (an inflection point range, marked in Fig. 6f, g), where the printed component could achieve its maximum SE_T_ and minimal SE_R_, resulting in the maximum absorption loss. Figure S11 shows the electromagnetic coefficients *A* and *R* of different dislocation structure, and the results show that the dislocation structure could positively enhance the absorption loss ratio of the material, with the absorption coefficient *A* being the largest near the critical inclined angle *θ*_*f*_. For the printed part with D^*^ = 2 mm, the absorption coefficient *A* is enhanced from 0.60 at *θ* = 0° to 0.76 at *θ*_*f*_; For the printed part with D^*^ = 4 mm, the absorption coefficient *A* increases from 0.66 at *θ* = 0° to 0.83 at *θ*_*f*_; also for the printed part with D^*^ = 6 mm, the absorption coefficient *A* increases from 0.38 at *θ* = 0° to 0.52 at *θ*_*f*_. The schematic diagram presented in Fig. 6h clarifies the underlying mechanism: (1) At *θ* < *θ*_*f*_, as *θ* measuring the degree of dislocation in pore increases, it would hinder the direct transmission of EMWs through these pores, thus effectively raising the SE_T_. Concurrently, the enlargement of *θ* would also enhance the multiple reflections and absorptions of EMWs inside the pores, thus further increasing the electromagnetic absorption of the printed part. Moreover, the larger D^*^ size, the more EMWs penetrated into the pores, rendering components more efficient of attenuating EMWs entering the pores. In contrast, for smaller D^*^ sizes, the losses of EMWs are predominant due to direct reflection and absorption on the product’s surface, with only a minimal amount of EMWs entering the pores. This would thereby minimize the impact of *θ* on the product’s shielding effectiveness. (2) At *θ* > *θ*_*f*_, where the pores are fully occluded, the change in the degree of dislocation hence has the minimal impact on the shielding effectiveness. With the continuous increase in *θ*, the degree of dislocation of the pores also increases, and the lower layers of pores would be gradually covered by the upper layers of pores deposited. As a result, in this case, a large number of EMWs reflected within the pores would leave the printed component directly, thus greatly weakening the absorption and reflection loss of EMWs within the pores. This would surely lead to a rise in SE_R_ value and a decrease in SE_T_ value. To sum up, such comprehensive exploration of the relationship between pore geometry, size, part thickness, and dislocation configuration with shielding effectiveness can provide the valuable experimental evidence and strategic guidance for the design and development of multi-porous EMI SE materials.

### 3D Printing of Highly Shielded Architected Honeycomb-Like Part

**Fig. 7:**
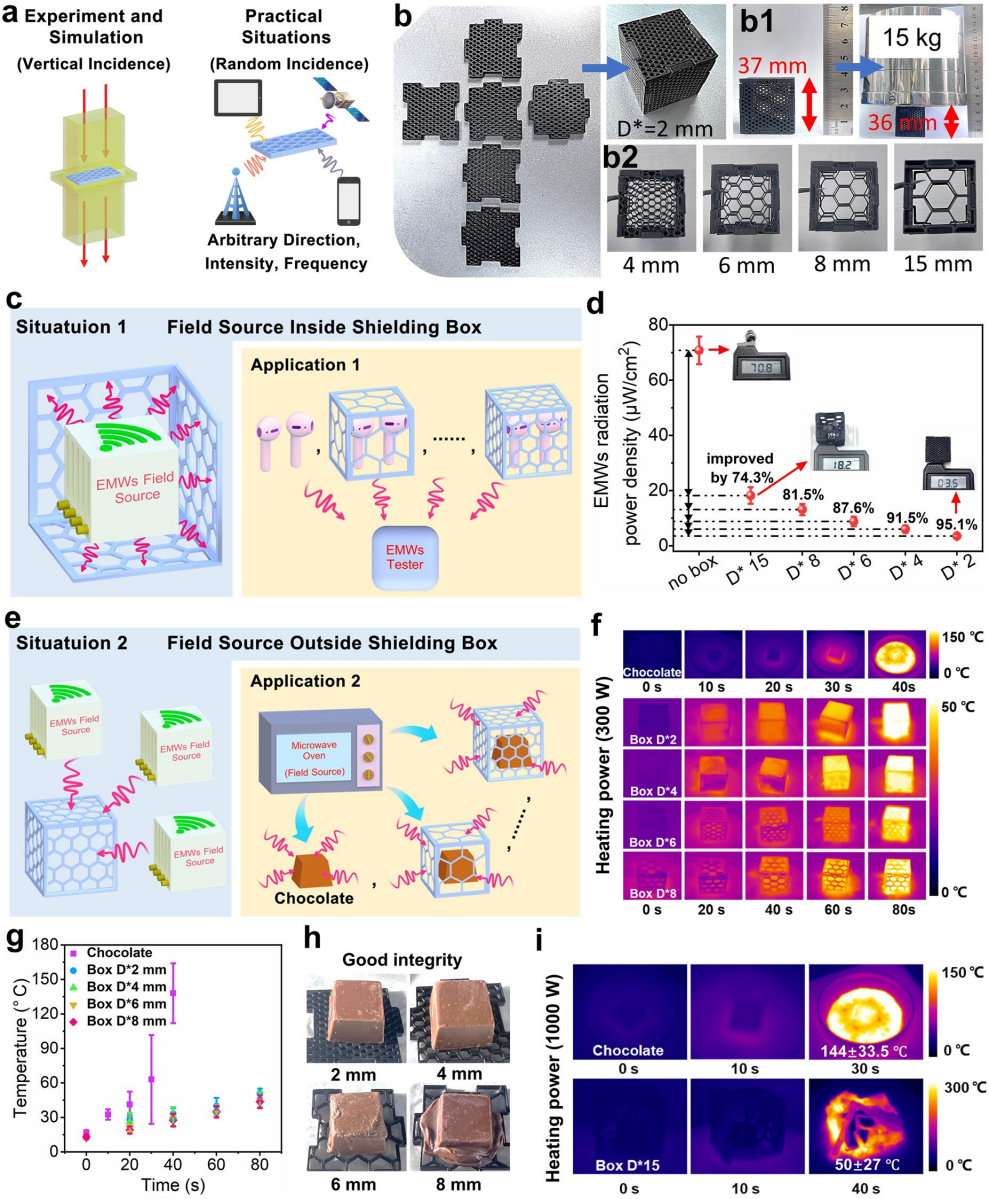
**a** Schematic diagram for various transmission form of electromagnetic waves in different scenarios; **b–b2** digital photographs of the printed shielding boxes with different D^*^ size and the compression test with a 15 kg load done on a D^*^ = 2 mm shielding box; **c** schematic diagram for placing earphone as EMWs field source inside the printed shielding box and **d** EMWs radiation intensity value received outside; **e** schematic diagram of using a microwave oven (300 W working power) as EMWs field source for microwave treatment of a printed shielding box with chocolate filled in, **f** the corresponding thermal imaging picture, **g** the extracted specific temperature values and **h** the digital photographs of the filled chocolate with microwave treatment; **i** the thermal imaging pictures of the naked chocolate and the filled chocolate in the printed D^*^ = 15 mm shielding box under irradiation of microwave oven with 1000 W power

As deeply discussed before, the relationship between the pore structures and the shielding effectiveness has been well developed in design and printing of porous EMI SE materials. However, the mentioned experiments and simulations are mainly based on the vertically incident plane waves, whereas in the real-world scenarios, EMWs sources are ubiquitous [57, 58]. This means that the waves of varying intensity, amplitude and phase will contact the shielding material at different angle of incidence (as shown in Fig. 7a). In light of these findings, in this section, various sizes of flexible honeycomb EMI SE boxes were designed and assembled to verify their shielding effectiveness in practical scenarios. Figure 7b–b2 shows the digital images of FDM 3D printed EMI shielding boxes and that of the mechanical test, exhibiting the clear unit structure, uniformity in size, excellent elasticity and good compression properties. Figures S12 and S13 show the compressive force versus strain curves and the EMI SE performance after compression of the box, respectively. It is seen that the mechanical and electromagnetic performance of the printed box could still be maintained after compression. (The detailed analyses are included in Supplementary Material.) This could allow them to be used for gap filling, providing improved filling and electrical continuity, and being directly attached to the external surfaces of devices to prevent the electromagnetic leakage and interference. Here, two interesting experiments were further demonstrated. In experiment 1 (Fig. 7c), the bluetooth earphone was employed as the electromagnetic field source (2.4 GHz), with a detector assessing the electromagnetic radiation intensity from the earphones in various shielding boxes. Figure 7d reveals that the radiation intensity of the earphones reached 70.8 μW cm^−2^. In contrast, after the working earphones were enclosed in the printed shielding boxes, there is a pronounced decrease in the radiation intensity detected outside the boxes. Obviously, it is found that the D^*^ = 15 mm shielding box with the largest pore size (*D*_*f*_) could attenuate the electromagnetic radiation to just 18.2 μW cm^−2^, a significant reduction of 74.3%. The D^*^ = 2 mm shielding box was found to be even more efficacious, reducing 95.1% of the electromagnetic radiation to a mere 3.5 μW cm^−2^. In experiment 2, as shown in Fig. 7e, a microwave oven with a power of 300 W was utilized as the electromagnetic wave source (2.45 GHz), and the tested chocolate was put inside the shielding box with different D^*^ size to evaluate the box’s shielding performance by observing the chocolate’s melting behavior. The infrared thermal imaging technique was accordingly applied to determine the temperature and temperature distribution of the chocolate treated by microwave. The obtained thermal imaging photographs and the extracted specific temperature values are shown in Fig. 7f, g, respectively. As can be seen, under an irradiation with 300 W electromagnetic power, the tested chocolate without any shielding succumbs to complete melting within only 40 s (Fig. 7f), with a temperature soaring to as high as 166 °C (Fig. 7g). In addition, the temperature rises sharply with the treating time. Comparatively, combining Fig. 7f, g, it is very clear that for the shielded chocolate, its temperature rises very slowly with time, and the degree of temperature rise decreases remarkably (in the shielding boxes with D* of 2, 4, 6, and 8 mm, with double treating time). For different D^*^ size, the difference in chocolate surface temperature is very small (the surface temperature could barely reach 50 °C). However, as seen from the appearance of the chocolate treated with 80 s (Fig. 7h), the difference in shielding effectiveness of different D^*^ size is remarkable, i.e., the shielding effectiveness of 2 and 4 mm is far better than that of 6 and 8 mm. For the shielding boxes with D^*^ of 2 and 4 mm, the chocolate sample could keep a good shape integrity, and however, for those with D^*^ of 6 and 8 mm, the partly melting behavior of chocolate sample can be observed, particularly for D^*^ of 8 mm. The above results clearly exhibit the high EMI shielding effectiveness of the designed printed shielding box. In order to further illustrate the highly shielding effect of the printed honeycomb-like part, the relatively less effective shielding box with bigger D^*^ size (15 mm) was used for demonstration under a much higher power (1000 W) for 40 s treating time. As a comparison, the naked chocolate was also put into the microwave oven and received an irradiation of microwave with the same power of 1000 W for a shorter treating time (30 s). The corresponding thermal imaging results are shown in Fig. 7i. As can be seen, in the absence of the printed shielding box, the chocolate rapidly melts within only 30 s, with a temperature soaring up to 177.5 °C. In contrast, under effect of the shielding box with D^*^ of 15 mm, even suffered the irradiation heating for 40 s, the chocolate inside the box remains intact and the surface temperature lies in the range of approximately 23–77 °C. The above result shows that even for the shielding box with a significantly enlarged D^*^ size (15 mm), it still could show a good EMI shielding role and effectively protect the chocolate from being irradiated under a significantly enhanced power of microwave. Obviously, the above experiments corroborate the printed shielding box’s capability to achieve the highly efficient broad-spectrum (> 2.4 GHz) shielding function, leveraging the tunable characteristics of the printed pores with the misaligned honeycomb-like unit structure.

## Conclusions

In order to overcome the significant challenges caused by the electromagnetic irradiation interference in some specific fields, in this study, we mainly focus on revealing and advancing the synergistic equilibrium correlation between porous structure and shielding efficiency based on design and realization of novel periodic porous structures through FDM 3D printing. A novel ultrasound-NIPS combination technique was firstly applied to fabricate the TPU/CNTs composite with a good uniformity and stability, achieving its maximum EMI SE efficiency of 46 dB. On this basis, thoroughly leveraging the advantage of FDM 3D printing strategy in forming the complex structures and shapes, it was performed to successfully print the periodic porous flexible shielding metamaterials with various pore shape, pore size, material thicknesses, and pore dislocation configuration. This study deeply explored the correlation between different periodic unit structure design and EMI SE efficiency by introducing a porosity factor *c*, which is the ratio of the inscribed (*D*_*in*_) circle diameter to the circumscribed (*D*_*out*_) one, thus revealing the hexagon-derived honeycomb structure as the most effective pore structure unit, with a shielding efficiency of 33–43 dB. In the subsequent pore size analysis, we eventually revealed and proposed the critical shielding failure size (*D*_*f*_) being in the range of *λ*/8 − *λ*/5; this *D*_*f*_ dimension provides guidance for tunable electromagnetic shielding of metamaterials. Based on determination of the *D*_*f*_ value, the careful regulation and optimization of the printed part thickness can further enhance the shielding efficiency and absorption coefficient *A* of the printed porous part, e.g., the maximum EMI shielding efficiency and absorption coefficient *A* could achieve 85–95 dB and over 0.87, respectively. In particular, the novel design of the pore dislocation configuration could further contribute a lot to the improvement of the overall shielding property and also the absorption loss. Finally, the printed porous EMI shielding parts were used to successfully and efficiently shield the working earphones and protect the chocolate from receiving the microwave irradiation and hence being melted, evidencing the high efficiency of the prepared periodic porous shields and showcasing the great potential of porous shields in mitigating the electromagnetic interference. This work demonstrates the feasibility of combining polymer composite with the advanced manufacturing technology to develop efficient EMI SE solutions, also offering new strategies and pathways for design and development of the flexible and highly efficient shielding metamaterials.

## Supplementary Information

Below is the link to the electronic supplementary material.

Supplementary file1 (DOCX 3400 KB)
