# Supplementary material for: 3D Printing of Periodic Porous Metamaterials for Tunable Electromagnetic Shielding Across Broad Frequencies
Source: Nanomicro Lett. 2024 Sep 3;16:279. doi: 10.1007/s40820-024-01502-5 (PMC11371985; doi:10.1007/s40820-024-01502-5)
Supplement: Supplementary file 1 — Supplementary file1 (DOCX 3400 KB) [file 40820_2024_1502_MOESM1_ESM.docx]

Supporting Information for

**3D Printing of Periodic Porous Metamaterials for Tunable Electromagnetic Shielding Across Broad Frequencies**

Qinniu Lv ^1^, Zilin Peng ^1^, Haoran Pei ^1^, Xinxing Zhang ^1^, Yinghong Chen ^1,^ *, Huarong Zhang ^2^, Xu Zhu ^2^, Shulong Wu ^2^

^1^ State Key Laboratory of Polymer Materials Engineering, Polymer Research Institute of Sichuan University, No. 24 South Section 1, Yihuan Road, Chengdu 610065, P. R. China

^2^ Baosheng Technology Innovation Corporation Limited, No. 1, Suzhong Road, Baoying County, Yangzhou 225800, P. R. China

* Corresponding author. E-mail: [johnchen@scu.edu.cn](mailto:johnchen@scu.edu.cn) (Yinghong Chen)

**S1 Experimental Part Related**

**Table S1** The sample numbers and compositions of TPU/CNTs nanocomposite with different filler loading

| Sample name | TPU (wt%) | CNTs (wt%) | PVP-K30 (wt%) | DMF (L) |
| --- | --- | --- | --- | --- |
| L-0.5 | 99.5 | 0.5 | 0.05 | 1 |
| L-1 | 99.0 | 1.0 | 0.1 |  |
| L-3 | 97.0 | 3.0 | 0.3 |  |
| L-6 | 94.0 | 6.0 | 0.6 |  |
| L-10 | 90.0 | 10.0 | 1.0 |  |

**Table S2** The printing processing parameters of the FDM 3D printer

| **Printing temperature** | **Platform**  **temperature** | **Layer**  **thickness** | **Printing**  **speed** | **Nozzle**  **diameter** |
| --- | --- | --- | --- | --- |
| 210 ^o^ C | 60 ^o^ C | 300 μm | 300 mm/min | 0.4 mm |

Due to the viscosity hysteresis behavior of the TPU material, the printing speed and nozzle diameter were selected at 300 mm/min and 0.4mm, respectively, to ensure the printing quality. The printing platform temperature was set at 60 ºC and the printing temperature was set at 210 ºC. To guarantee the sufficient interface adhesion between filaments, the layer thickness was set at 300 μm.

**S2 Results & Discussion Related**

In the ultrasonic dispersion stage for CNTs, an evaluation of the stability in CNTs suspensions under different dispersion conditions was performed (Fig. S1). The optimal conditions were determined to be a combination of ultrasonication, mechanical stirring, and use of a dispersant.

| **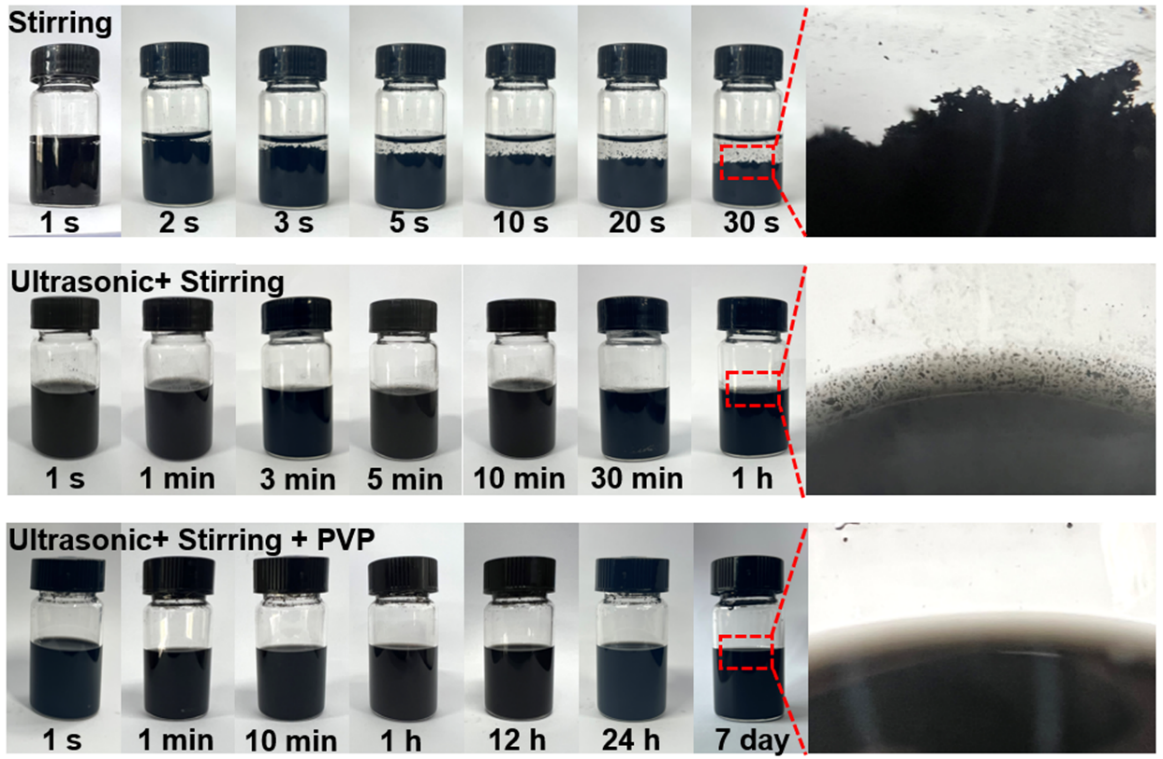** |
| --- |
| **Fig. S1** Optical images of CNTs under different dispersion conditions |

Figure S2 show the fractured cross-section morphology of the finally obtained TPU/CNTs nanocomposite material, revealing that the CNTs still exhibit the outstanding dispersion property. This is due to the rapid exchange between DMF and water during the solvent exchange process, which could enable the rapid precipitation and formation of the TPU/CNTs compound. Such the quick process could prevent the CNTs from being agglomerated and settling, thus preserving their original dispersion state in the suspension to a great extent, as illustrated in Fig. S3.

**
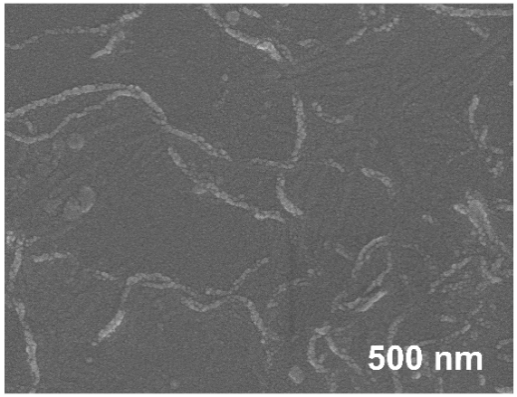
**

**Fig. S2** SEM image of cross section of TPU/CNTs composite

**
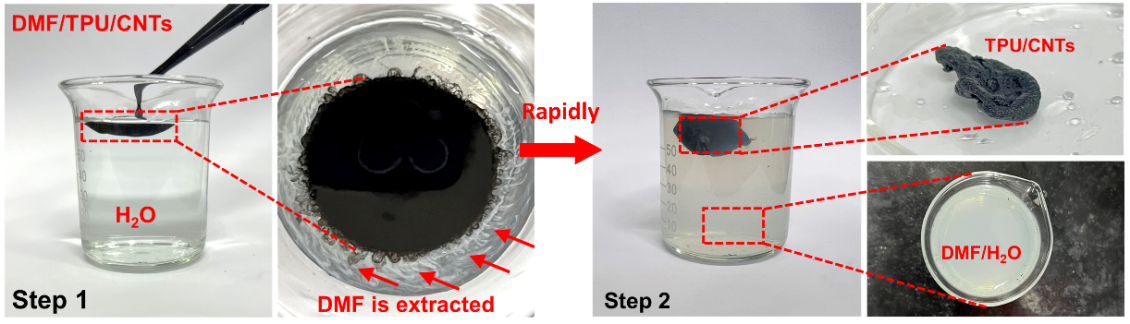
**

**Fig. S3** Optical image of NIPS process

The appearance and the fractured cross-section of the TPU/CNTs filament were characterized by SEM observation and the results are shown in Figs. S4-S5. It can be seen that the obtained TPU/CNTs filaments show a smooth surface and a uniform diameter of 1.75 mm, which completely meet the requirements for FDM 3D printing. Very clearly, the cross-section surface roughness of the filaments incrementally rises with increase in CNT content, and yet the filament surface is smooth on the whole, which is devoid of any CNT agglomerations or stress concentration points.

| **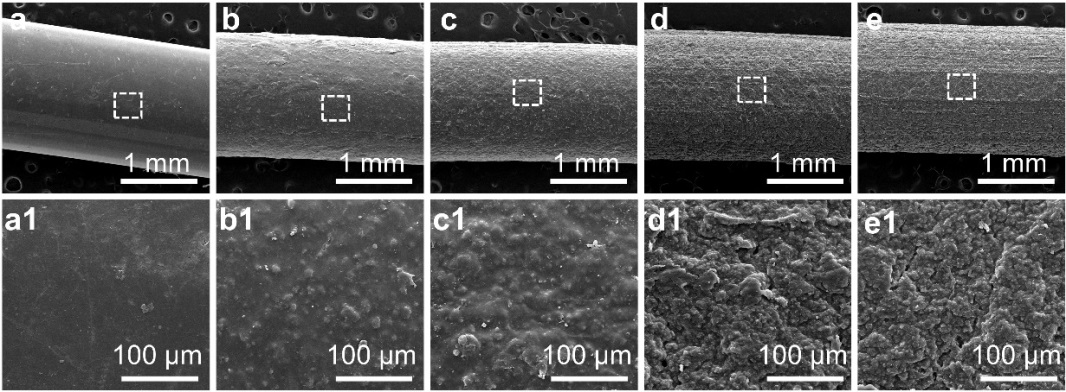** |
| --- |
| **Fig. S4** SEM images of the surface of L-n filaments, numbers a-e are L-0.5, L-1, L-3, L-6, and L-10, respectively |
| **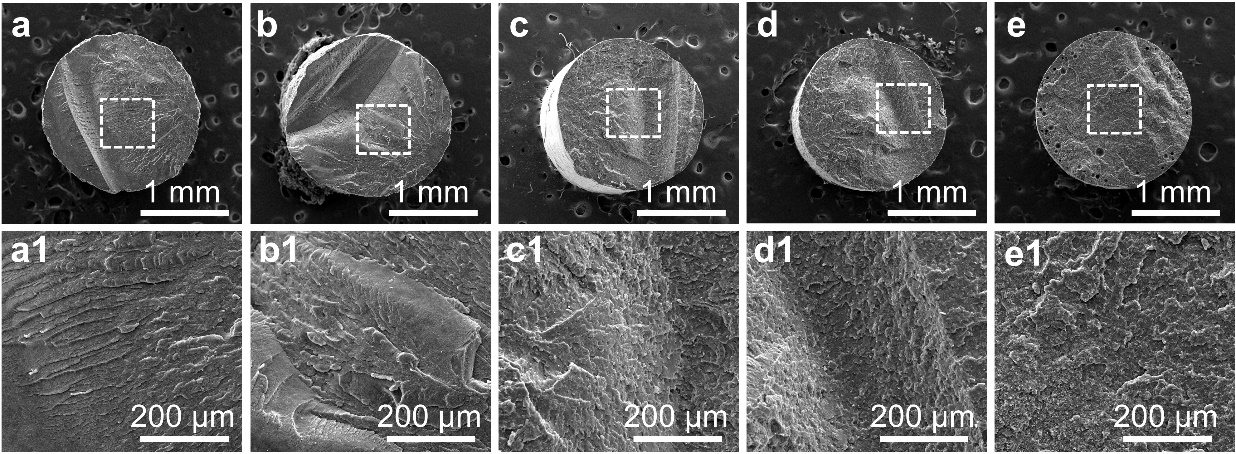** |
| **Fig. S5** SEM images of the cross-section of L-n filaments, numbers a-e are L-0.5, L-1, L-3, L-6, and L-10, respectively |

Rheological data provide a clear depiction of CNTs distribution in the TPU matrix, where the influence of frequency on both the storage modulus *G'* and the loss modulus *G''* is consistent across all samples, especially notable in the high-frequency domain (refer to Figs. S6a-b). In the lower frequency range, the L-n samples show a plateau phase, suggesting the gradual formation of physical junctions by CNTs within the TPU, which suppresses the relaxation behavior of TPU molecular chains, thereby diminishing their frequency dependence. For L-6, L-10 with higher CNTs loading, the dispersion of CNTs throughout the TPU interior significantly increases the interface area between CNTs and TPU, further inhibiting the movement of molecular chains, leading to the continuous refinement of the filler network in the L-n composites.

| **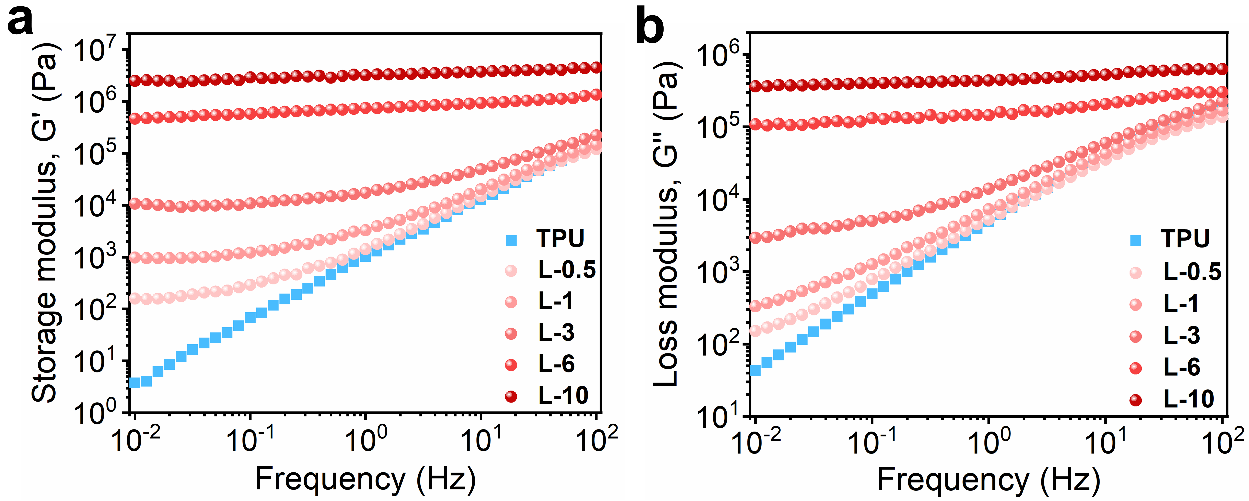** |
| --- |
| **Fig. S6** Frequency dependences of **a** storage modulus (*G′*) and **b** loss modulus (*G″*) for L-n composites with different CNTs content |

As depicted in Fig. S7, the conductivity of the L-n composites undergoes a pronounced transformation beyond L-0.5, signifying the establishment of a conductive network. With the increment in CNTs concentration, the electrical conductivity of the L-n materials increases. This conductivity data has been analyzed using the power law equation based on percolation theory [S[1](#_ENREF_1)]:

$\sigma=\sigma_{0}{(\varphi-\varphi_{c})}^{t} (\varphi>\varphi_{c})$ (S1)

where *σ* represents the conductivity, *σ_0_* is a constant reflecting the intrinsic conductivity of G, *φ* is the volume fraction of GNPs, *φ_c_* is the percolation threshold, and *t* is a critical exponent revealing the dimensionality of the conductive networks. The analysis reveals that the electrical percolation threshold of the L-n samples stands at merely 0.975 wt%, indicating that an increment in CNTs leads to the development of a robust and stable conductive network within the L-n matrix.

| 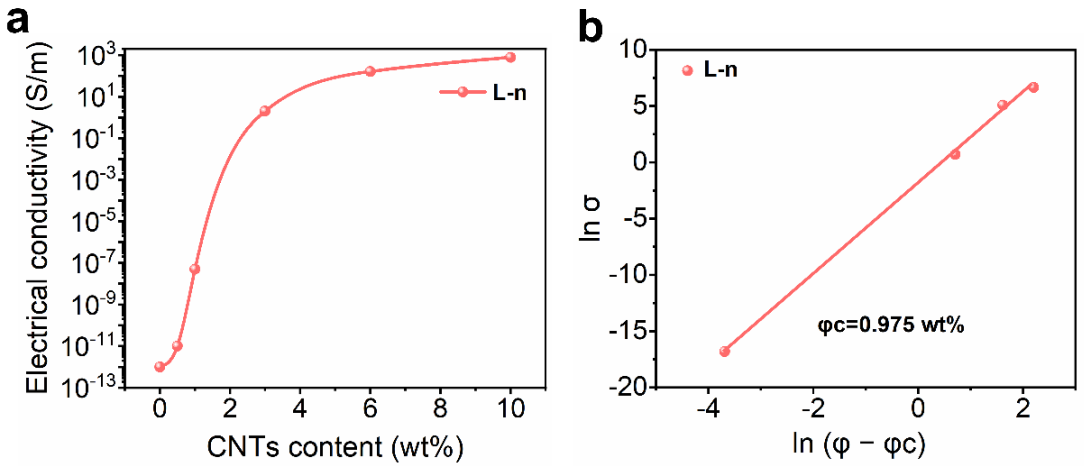 |
| --- |
| **Fig. S7 a** Electrical conductivity and **b** fitted curve of L-n composites with different CNTs content |

As shown in Fig. S8, the linear pore corresponds to *c*=0, triangular pore corresponds to *c*=0.5, square pore corresponds to *c*=0.71, hexagonal pore corresponds to *c*=0.87, and circular pore corresponds to *c*=1, respectively. Obviously, the *c* value can well and completely reflect the unit pore structure of the printed metamaterials as a structural parameter. Generally, *D_in_* can only normalize the size of the pores and does not represent the structural difference between the four different pores well. Comparatively, the pore factor *c* can well embody the relationship between different type of pores in a normalized way (*c* = *D_in_*/*D_out_* (0 ≤ *c* ≤ 1)). Therefore, the *c*-value could help to understand the effect of the above different pore structure on the EMI SE property and also the integrity of the involved eddy current.

| 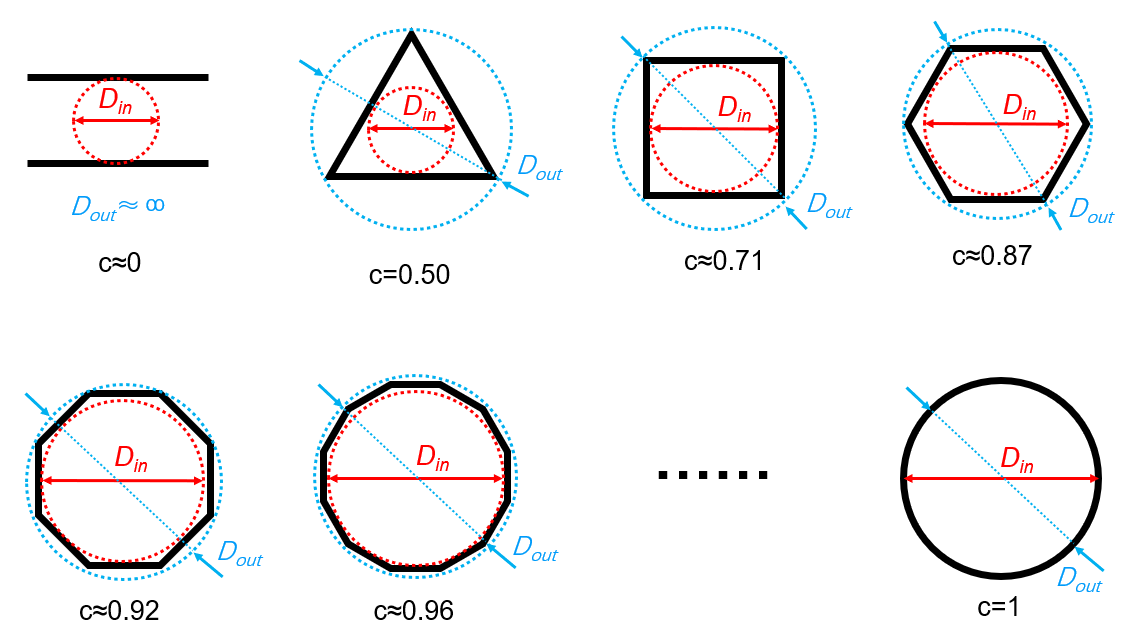 |
| --- |
| **Fig. S8** The schematic diagram of pore factor *c* with different pore structure |

Here, the influence of different pore structure on the induced eddy currents and the induced electric fields is illustrated deeply and detailedly. The absorption loss of the shielding body is due to the Ohmic heat loss caused by induced eddy currents. Meanwhile, the reverse magnetic field generated by eddy currents would offset some of the original magnetic fields. As a result, ensuring the unobstructed flow of eddy currents is very important in maintaining good shielding effectiveness. If there is a gap in the shielding material, which is perpendicular to the direction of the eddy current, the eddy current would be blocked and can only bypass the gap, as shown in Fig. S9. According to the electromagnetic field theory, the gap is equivalent to a secondary transmitting antenna, emitting energy into the shielding body, greatly reducing the shielding effectiveness of the shielding body. If the gap length direction is parallel to the direction of eddy current, the eddy current has less impact, so the gap length is an important factor in determining the shielding effectiveness of the shielding body. Although in practice it is not possible to predict the location and magnitude of the eddy current, the trajectory of the eddy current flow can be regarded as a circle of vortex. So, in order to reduce the damage to the eddy current, the only way is to minimize the length of the gap or to make the pore structure be closer to a circle. As a result, it is very clear that the linear, triangular and square structures can severely damage the integrity of eddy currents in their corner/edge regions, resulting in a decrease in shielding effectiveness. In addition, the parameter of Power loss density in the simulations (Fig. 3g) was also used to quantify the electromagnetic power dissipated within lossy materials. It can be seen that the Power loss density tends to be almost blank in the linear structure, implying that the linear structure is almost incapable of blocking EMWs. In the case of the triangular and square structures, there is a significant loss seen only in the corner areas (or perpendicular to the EMWs incident direction), which means that the sharper corner areas could effectively block the eddy currents. The honeycomb structure is closest to the vortex structure of eddy currents, and hence the resultant loss of EMWs is uniform and efficient.

It should be noted that the reason why we used the parameter *c* in this paper is that it could effectively represent the relationship of all the pores in a uniform way (*c* = *D_in_*/*D_out_* (0 ≤ *c* ≤ 1)). The variation of the *c* value represents the transition of pore structure from linear (*c*=0) to triangular (*c*=0.5), square (*c*=0.71), hexagonal (*c*=0.87), and circular (*c*=1), respectively. Therefore, the *c*-value helps to understand the effect of the above pore structure on the integrity of the eddy current. The smaller the *c*-value, the larger the aspect ratio of the pore structure, and the easier it can destroy the integrity of the eddy current; the larger the *c*-value, the closer it would be to a circular shape, and the better it could maintain the integrity of the eddy current.


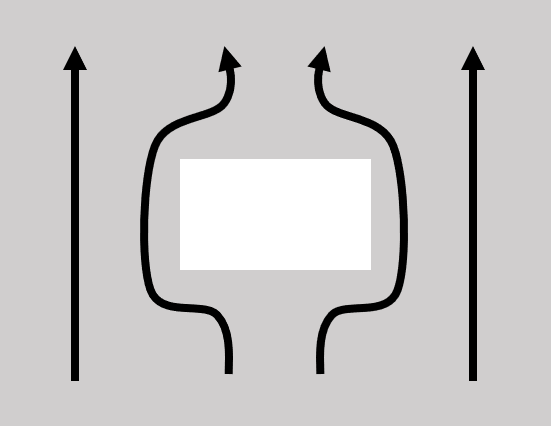


**Fig. S9** Schematic diagram of the gap perpendicular to the direction of the eddy currents and this will cause the eddy current to be blocked and only bypass the gap

| 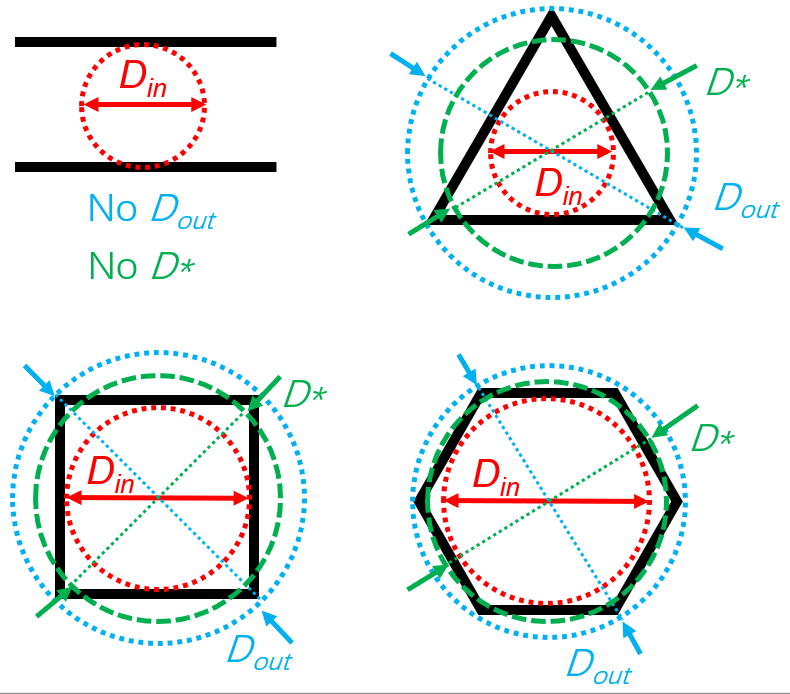 |
| --- |
| **Fig. S10** The *D** and *D_in_* and *D_out_* of different unit pore structure |

The reason why the critical failure size *D_f_* increases with increasing the printed component thickness is analyzed as follows: combining with the schematic diagram shown in Fig. 4h, at the same equivalent diameter *D^*^*, the printed parts with smaller thickness would have the less loss of EMWs inside the pores, and however, more amount of the EMWs would easily pass through the pore directly, resulting in a decrease in EMI SE effectiveness; while with the increase in thickness of the printed part, the proportion of EMWs absorbed and reflected inside the pores would increase substantially, and it is difficult for the EMWs to pass through the pores directly, which would thus lead to the increase in *D_f_* size with the increase in thickness.





**Fig. S11** The absorption coefficient *A* and reflection coefficient *R* of printed samples with different inclined angle *θ* (dislocation structure)

It should be noted that the shielding box has a three–dimensional size of 37.0*37.0*37.0 mm, which is assembled with 6 shielding plates, and each shielding plate has a large number of pores. As a result, its compression cross-section area during compression is difficult to calculate. Therefore, we performed the compression test in a form of compression force vs strain curve, but not compression strength vs strain curve (Fig. S12). As can be seen, the smaller the pore size of the shielding box, the greater the compressive force, and each printed sample could achieve a maximum compression force of about 470 N (BOX D*2), 370 N (BOX D*4), 260 N (BOX D*6), 195 N (BOX D*8), and 80 N (BOX D*15), respectively. In addition, since the shielding box is directly assembled without any reinforcement measures adopted, when the compression deformation of the shielding box reaches around 5-9%, the assembled surfaces will be disintegrated or collapse. As a result, the maximum compression force and the maximum strain mentioned above are not the limits of the materials and the honeycomb structures themselves however. If the appropriate reinforcement methods are adopted to make the joint surfaces tightly connected, the maximum compression force and the maximum deformation of the box could be significantly enhanced.

|  |
| --- |
| **Fig. S12** The compressive force versus strain curves of shielding box with different pore size |

As mentioned above, the shielding box is an elastomeric composite material. Obviously, a deformation of only 5-9% would not cause the permanent deformation of material, and it would not also change the filler distribution in the polymer matrix, so the electromagnetic shielding effectiveness of the printed sample would not be changed theoretically. In order to prove the above idea, we compressed a 3D printed part of 22.9*10.2*4.0 mm dimension featuring aperture size *D^*^*=2.0 mm in a 10% deformation along the three directions of length (X), width (Y), and height (Z). The results show that the electromagnetic shielding effectiveness basically does not change (Fig. S13).

| 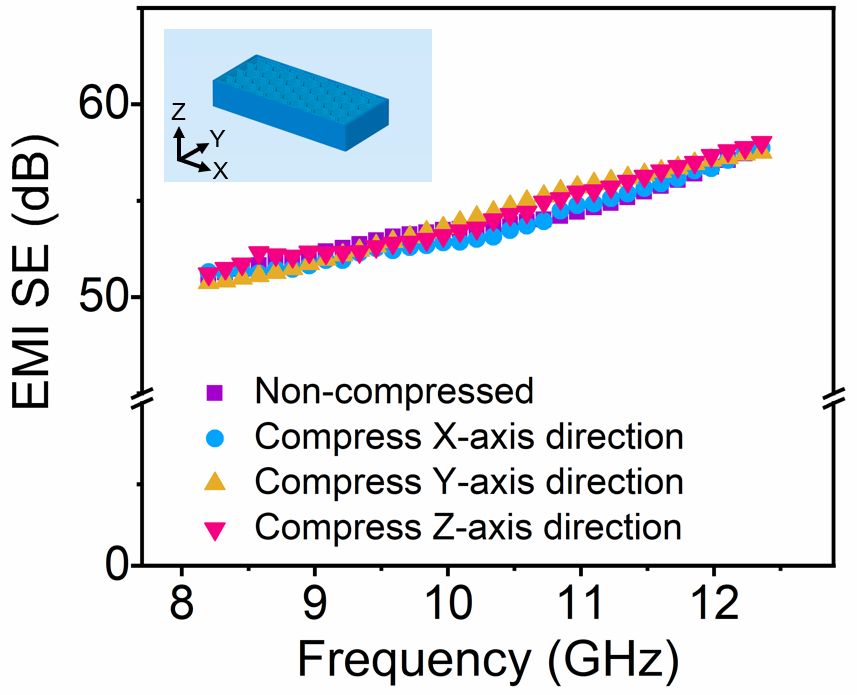 |
| --- |

**Fig. S13** The EMI SE results of the printed parts with dimension of 22.9×10.2×4.0 mm and pore size *D^*^*=2.0 mm after compression in different direction

The electromagnetic simulations were performed on the 3D printed components with 4 different structures using CST Studio Suite software. The related simulation parameters are included in Table S3 and the detailed simulation processes could include the following steps: (1) defining the electromagnetic field engineering template (including microwaves & RF/optical, periodic structures, FSS metamaterial-unit cell, phase reflection diagram, frequency domain and other parameters); (2) defining the material parameters (the material used in this work is L-10, which is defined using the conductivity or electromagnetic parameters of L-10 material); (3) constructing the material model (structural parameters of the pore structure); (4) defining the background and boundary of the electromagnetic field; (5) choosing the parameters to be simulated (except for S-parameters, the “E-field” and “Power loss density” are also considered in this work, where “Power loss density” is a parameter of CST simulation software used to quantify the electromagnetic power dissipated in the loss material).

**Table S3** The simulation parameters used in this work

| Sample | straight | triangle | square | | honeycomb | |
| --- | --- | --- | --- | --- | --- | --- |
| Project Solver | Frequency Domain | | | | | |
| Dimensions | mm | | | | | |
| Frequency | 8.2-12.4 GHz | | | | | |
| EMWs Mode | TE10 | | | | | |
| Boundary Conditions | X/Y: unit cell，Z: open | | | | | |
| Background | Air (loss free) | | | | | |
| Material Parameter | The parameters are the same as L-10 | | | | | |
| Modeling Parameters | Brick | Cylinder | | | | |
|  | X min: -*a*/2  X max: *a*/2  Y min: -*d*/2  Y max: *d*/2  Z min: 0  Z max: *L*  Y-axis translation distance: *r* | Segments: 3  Outer radius: 2*(*r*+*d*)  inner radius: 2**r*  Z min: 0  Z max: *L* | | Segments: 4  Outer radius: (*r*+*d*)*sqr(2)  inner radius: *r**sqr(2)  Z min: 0  Z max: *L* | | Segments: 6  Outer radius: (*r*+*d*)*sqr(3)*2/3  inner radius: *r**sqr(3)*2/3  Z min: 0  Z max: *L* |
|  | *a* (The length of a straight line): ∞;  *d* (Nozzle diameter): 0.4;  *r* (The size of *D_in_*): 2  *L* (Sample thickness): 2 | | | | | |

Table S4 illustrates the correlation between *D^*^*, *D_in_*, and *D_out_* across various structures. It is observed that *D^*^* is intermediate between *D_in_* and *D_out_*, the closer the pore structure is to a circular shape, the better the *D** reflects the true size of the pore structure. Thus, *D_in_* is more suitable for qualitatively comparing pores of different structures, whereas *D^*^* is more suitable for representing the dimensions of a single type of hole, such as a honeycomb-type pore.

**Table S4** The relationship between *D^*^* of different pore structure and their *D_in_* and *D_out_*

| **Pore units** | ***D_in_*** | ***D_out_*** |
| --- | --- | --- |
| Straight | *D_in_* | / |
| Triangle | 0.63 *D** | 1.26 *D** |
| Square | 0.82 *D** | 1.16 *D** |
| Honeycomb | 0.92 *D** | 1.06 *D** |

**Table S5** The comparison of this work with other literatures involving carbon-based functional materials in EMI SE performance [S[2-S13](#_ENREF_2)].

| **Composites** | **Filler contents (wt %)** | **Thickness (mm)** | ***ρ* (g/cm3)** | **EMI SE (dB)** | **Refs.** |
| --- | --- | --- | --- | --- | --- |
| TPU/CNTs | 10 | 2 | 0.44 | 38.7 | This work |
|  |  |  | 0.47 | 39.3 |  |
|  |  |  | 0.65 | 40.9 |  |
|  |  |  | 0.78 | 42.5 |  |
|  |  |  | 0.92 | 42.7 |  |
|  |  |  | 0.96 | 44.3 |  |
|  |  |  | 1.00 | 44.2 |  |
|  |  |  | 1.06 | 45 |  |
|  |  |  | 1.10 | 47 |  |
| POE/GNPs | 30 | 2 | 0.65 | 25 | [S2] |
|  |  |  | 0.75 | 26 |  |
|  |  |  | 0.86 | 30 |  |
|  |  |  | 0.96 | 31 |  |
|  |  |  | 1.06 | 32 |  |
| PLA/GNPs/CNTs | 8 | 2 | 0.47 | 32 | [S3] |
|  |  |  | 0.62 | 36 |  |
|  |  |  | 0.82 | 10 |  |
| TPU/CNTs | 5 | 2 | 1.22 | 35 | [S4] |
| SR/MWCNTs | 10 | 2 | 0.44 | 25 | [S5] |
|  |  |  | 0.49 | 27 |  |
|  |  |  | 1.04 | 10 |  |
|  |  |  | 1.07 | 30 |  |
|  |  |  | 1.13 | 15 |  |
| PS/MWCNTs | 5 | 2 | 0.96 | 17 | [S6] |
| PS/MWCNTs | 7 | 1.8 | 0.47 | 23 | [S7] |
|  |  |  | 0.60 | 26 |  |
| PVDF/CNTs/ Gr | 16 | 2 | 0.53 | 10 | [S8] |
|  |  |  | 0.55 | 12 |  |
|  |  |  | 0.59 | 18 |  |
|  |  |  | 0.62 | 27 |  |
| PMMA/ Gr | 5 | 3.4 | 1.19 | 25.5 | [S9] |
| NR/Fe3O4/rGO | 10 | 1.88 | 0.93 | 34 | [S10] |
| PMMA/ Gr | 5 | 2.4 | 0.79 | 19 | [S11] |
| PU/MWCNTs | 10 | 1.5 | 1.23 | 18 | [S12] |
| PTT/MWCNTs | 10 | 2 | 1.3 | 23 | [S13] |

Note: GNPs: graphene nanosheets; CNTs: carbon nanotubes; MWCNTs: multiwalled carbon nanotubes; Gr: graphene; rGO: reduced graphene oxide; POE: polyolefin elastomer; PVDF: polyvinylidene fluoride; PLA: polylactic acid; NR: natural rubber; SR: silicone rubber; PTT: poly (trimethylene terephthalate); PMMA: polymethyl methacrylate; PU: polyurethane; PS: polystyrene.

**Supplementary References**

1. B. Wei, L. zhang, S. Yang, Polymer composites with expanded graphite network with superior thermal conductivity and electromagnetic interference shielding performance. Chem. Eng. J. **404**, 126437 (2021). <https://doi.org/10.1016/j.cej.2020.126437>
2. Q. Lv, Z. Peng, Y. Meng, H. Pei, Y. Chen et al., Three-dimensional printing to fabricate graphene-modified polyolefin elastomer flexible composites with tailorable porous structures for electromagnetic interference shielding and thermal management application. Ind. Eng. Chem. Res. **61**, 16733-16746 (2022). <https://doi.org/10.1021/acs.iecr.2c03086>
3. Q. Lv, X. Tao, S. Shi, Y. Li, N. Chen, From materials to components: 3D-printed architected honeycombs toward high-performance and tunable electromagnetic interference shielding. Compos. Part B: Eng. **230**, 109500 (2022). <https://doi.org/10.1016/j.compositesb.2021.109500>
4. D. Feng, D. Xu, Q. Wang, P. Liu, Highly stretchable electromagnetic interference (EMI) shielding segregated polyurethane/carbon nanotube composites fabricated by microwave selective sintering. J. Mater. Chem. C **7**, 7938-7946 (2019). <https://doi.org/10.1039/c9tc02311a>
5. J. Yang, X. Liao, J. Li, G. He, Y. Zhang et al., Light-weight and flexible silicone rubber/MWCNTs/Fe3O4 nanocomposite foams for efficient electromagnetic interference shielding and microwave absorption. Compos. Sci. Technol. **181**, 107670 (2019). <https://doi.org/10.1016/j.compscitech.2019.05.027>
6. M. Mahmoodi, M. Arjmand, U. Sundararaj, S. Park, The electrical conductivity and electromagnetic interference shielding of injection molded multi-walled carbon nanotube/polystyrene composites. Carbon **50**, 1455-1464 (2012). <https://doi.org/10.1016/j.carbon.2011.11.004>
7. J. Chen, X. Liao, W. Xiao, J. Yang, Q. Jiang et al., Facile and green method to structure ultralow-threshold and lightweight polystyrene/MWCNT composites with segregated conductive networks for efficient electromagnetic interference shielding. ACS Sustain. Chem. Eng. **7**, 9904-9915 (2019). <https://doi.org/10.1021/acssuschemeng.9b00678>
8. X. Ma, B. Shen, L. Zhang, Y. Liu, W. Zhai et al., Porous superhydrophobic polymer/carbon composites for lightweight and self-cleaning EMI shielding application. Compos. Sci. Technol. **158**, 86-93 (2018). <https://doi.org/10.1016/j.compscitech.2018.02.006>
9. H. Zhang, W. Zheng, Q. Yan, Z. Jiang, Z. Yu, The effect of surface chemistry of graphene on rheological and electrical properties of polymethylmethacrylate composites. Carbon **50**, 5117-5125 (2012). <https://doi.org/10.1016/j.carbon.2012.06.052>
10. Y. Zhan, J. Wang, K. Zhang, Y. Li, Y. Meng et al., Fabrication of a flexible electromagnetic interference shielding Fe_3_O_4_@ reduced graphene oxide/natural rubber composite with segregated network. Chem. Eng. J. **344**, 184-193 (2018). <https://doi.org/10.1016/j.cej.2018.03.085>
11. H. Zhang, Q. Yan, W. Zheng, Z. He, Z. Yu, Tough graphene-polymer microcellular foams for electromagnetic interference shielding. ACS Appl. Mater. Inter. **3**, 918-924 (2011). <https://doi.org/10.1021/am200021v>
12. T. Gupta, B. Singh, S. Dhakate, V. Singh, R. Mathur, Improved nanoindentation and microwave shielding properties of modified MWCNT reinforced polyurethane composites. J. Mater. Chem. A **1**, 9138 (2013). <https://doi.org/10.1039/c3ta11611e>
13. A. Gupta, V. Choudhary, Electrical conductivity and shielding effectiveness of poly(trimethylene terephthalate)/multiwalled carbon nanotube composites. J. Mater. Sci. **46**, 6416-6423 (2011). <https://doi.org/10.1007/s10853-011-5591-8>
